# Circular RNAs exhibit exceptional stability in the aging brain and serve as reliable age and experience indicators

**DOI:** 10.1016/j.celrep.2025.115485

**Published:** 2025-04-02

**Authors:** Ken Kirio, Ines Lucia Patop, Ane Martin Anduaga, Jenna Harris, Nagarjuna Pamudurti, The Nandar Su, Claire Martel, Sebastian Kadener

**Affiliations:** 1 Biology Department, Brandeis University, Waltham, MA 02454, USA; 2 Lead contact

## Abstract

Circular RNAs (circRNAs) increase in the brain with age across various animal systems. To elucidate the reasons behind this phenomenon, we profile circRNAs from fly heads at six time points throughout their lifespan. Our results reveal a linear increase in circRNA levels with age, independent of changes in mRNA levels, overall transcription, intron retention, or host gene splicing, demonstrating that the age-related accumulation is due to high stability rather than increased biogenesis. This remarkable stability suggests that circRNAs can serve as markers of environmental experience. Indeed, flies exposed to a 10-day regimen at 29°C exhibit higher levels of specific circRNAs even 6 weeks after returning to standard conditions, indicating that circRNAs can reveal past environmental stimuli. Moreover, half-life measurements show circRNA stability exceeding 20 days, with some displaying virtually no degradation. These findings underscore the remarkable stability of circRNAs *in vivo* and their potential as markers for stress and life experiences.

## In brief

Kirio et al. demonstrate that circRNAs steadily accumulate in the aging fly brain due to their exceptional stability. Temperature exposure (29°C) further elevates select circRNAs, which remain high weeks later, underscoring their potential as markers of both age and past experiences.

## Graphical Abstract

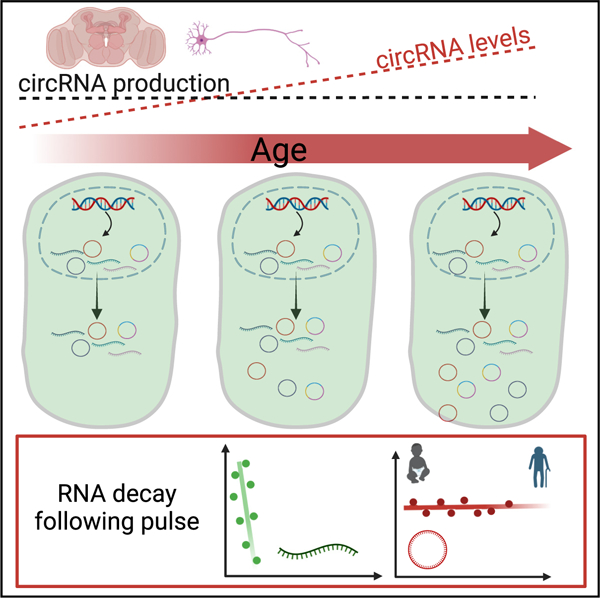

## INTRODUCTION

Circular RNAs (circRNAs) are a large class of stable RNAs produced through the circularization of specific exons.^1–8^ They are generated by the spliceosome via “backsplicing” in which the 3′ end of an exon is covalently linked to the 5′ end of the same or an upstream exon.^1,9,10^ CircRNA biogenesis depend on *cis*-elements, such as inverted complementary sequences in the flanking introns^11–13^ and *trans-*acting factors such as the splicing factors MBL, QUAKING, NOVA2, and others.^9,14–16^ As circRNAs are generated co-transcriptionally and in competition with linear splicing, their production can regulate gene expression in *cis*.^9,17^ Furthermore, recent studies have identified *trans* functions for specific circRNAs; for example CDR1as regulates miR-7^5,6,18^ and some circRNAs sequester or transport proteins, regulate ribosomal RNA (rRNA) biogenesis, or play roles in development.^9,19–23^ CircRNAs also mediate responses to viral infections.^24–27^ Additionally, research from various groups, including ours, has shown that some circRNAs can produce proteins.^28–31^

CircRNAs are highly enriched in the brain, with some also enriched in synapses and regulated by neuronal activity.^32–35^ Since circRNA levels inversely correlate with cell replication rates,^36^ the high levels of these molecules in neural tissues likely reflect their post-mitotic status. Furthermore, the prevalence of alternative splicing in neurons may contribute to the extensive diversity of circRNAs in the brain.^37,38^ CircRNAs exhibit evolutionary conservation; for example, 80% of mouse circRNAs with moderate-to-high expression levels in the brain are also present in the human brain.^33^ Indeed, several circRNAs regulate synaptic function and behavior. For instance, knockout (KO) of CDR1as in mice results in behavioral defects,^39^ knockdown (KD) of circMbl in *Drosophila* leads to locomotor activity abnormalities,^17^ circSLC45A4 is essential for progenitor state maintenance in the mouse brain,^40^ and KO of circTulp4 leads to severe defects in neuronal transmission in mice.^41^ Recent studies suggest functions of circRNAs in aging. For example, circSfl and circ-crh-1 control lifespan and aging in *Drosophila*^42^ and *Caenorhabditis elegans*,^43^ respectively, and several circRNAs regulate senescence in mammalian cell culture.^22,44^ Additionally, circRNAs are dysregulated in various diseases, including cancer, amyotrophic lateral sclerosis (ALS), and Alzheimer and Parkinson’s disease.^19,45–47^ Despite these insights, the functions of thousands of circRNAs remain to be elucidated.^48^

CircRNAs evade degradation by canonical mRNA-turnover pathways,^49^ and studies in cell culture have demonstrated their high stability, with half-lives ranging from 12 to 16 h to several days.^9,50–52^ However, no studies determining the stability of circRNAs have been performed *in vivo.* In addition, research across multiple systems has shown that overall circRNA levels increase with age in the brains of mice, worms, and flies,^35,53–55^ as well as in specific human brain regions.^45^ However, it remains unknown whether this age-dependent increase results from low degradation rates and/or an increase in the biosynthesis of circRNAs. Understanding this process might have significant implications for aging, as some evidence suggests that the circRNA age-dependent accumulation can be toxic. For example, knocking out a circRNA that dramatically increases with age (*crh-1* circRNAs) extends lifespan in *C. elegans*^43^ and provides protection in animal models of Alzheimer’s disease.^56^ As previously mentioned, circRNAs exhibit extreme stability. This stability along with their cell-specific expression and dysregulation in disease prompted the use of circRNAs as disease biomarkers.^7,8,49^ Additionally, the documented regulation of circRNAs by certain stresses led us to propose that circRNAs could serve as life experience markers (or “flight recorders”^49^). However, this hypothesis has not yet been tested.

In this study, we profiled circRNAs in the heads of male and female flies at six time points across their lifespan. We found a linear, age-dependent increase in global circRNA levels, making circRNAs superior age markers compared with linear mRNAs. Individual circRNAs also accumulate with age at different rates, and this does not stem from changes in transcription, splicing, or precursor production, indicating that high circRNA stability drives their accumulation. Exposing flies to low (18°C) or high (29°C) temperatures for 10 days induced specific subsets of circRNAs, which remained elevated even after return to standard conditions for several weeks. Furthermore, half-life measurements show very low degradation in fly neural tissue, with some circRNAs likely not degraded at all. Thus, the age-dependent accumulation of circRNAs in neurons reflects their intrinsic stability, allowing them to serve as stress/life experience markers.

## RESULTS

### Overall circRNA levels increase continuously with age in fly heads

**Figure 1.**
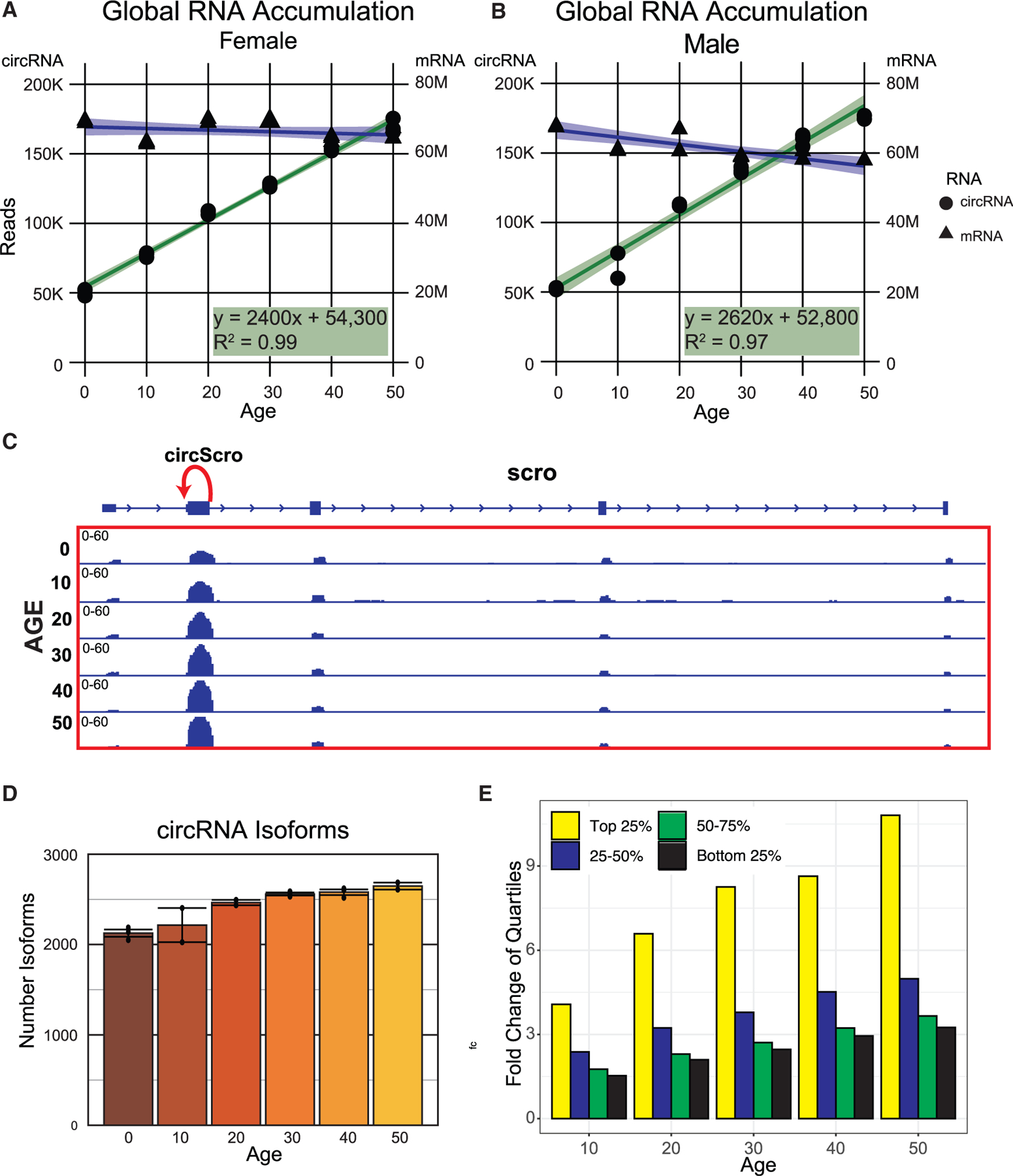
circRNAs increase linearly with age in *Drosophila* heads (A) Total number of DESeq2 normalized circRNA and linear reads at each time point across life in female flies, represented by green/circles and blue/triangles, respectively. The circRNA reads fit a linear regression with an R^2^ value of 0.99. (B) Similar to (A), but for male flies. The circRNA reads fit a linear regression with an R^2^ value of 0.97. (C) IGV snapshot in the *scro* gene showing the position of the major circScro isoform (red arrow) and the noticeable increase in the exon signal containing this circRNA as the fly ages. (D) Number of different circRNA isoforms in male flies as they age. Plotted are means ± SEM. (E) Fold change in expression levels (compared with the first time point) for backsplicing reads corresponding to circRNAs in each expression quartile in females.

Previous work showed that circRNAs increase with age in neuronal tissues in flies, worms, mice, and some human brain regions.^35,45,53,54^ However, these experiments utilized in most cases limited time points and/or suboptimal sequencing depth and length, which compromised comprehensive detection of circRNAs. To address this limitation, we conducted RNA sequencing (RNA-seq) on fly head samples collected at 10-day intervals across a span from newly eclosed (denoted as 0 for simplicity) to 50 days of age. We carried out separate experiments for male and female flies, with three samples collected for most age groups. The RNA-seq libraries were sequenced using 150-base paired-end reads at a depth of at least 50–60 million reads *per* sample (resulting in >160 million aligned pair-end reads *per* time point *per* sex on average, Table S1). Following quality control and removal of two outlier samples, we identified nearly 29,000 circRNAs. A majority of these circRNAs are likely to be splicing artifacts or sequencing abnormalities, as over 60% of them do not span any annotated splice junction (Figure S1A) and are either present in only one sample or detected at very low levels. Subsequently, we applied stringent filters to exclude lowly expressed and low-confidence circRNAs, resulting in a refined list of 2,866 high-confidence circRNAs (Table S2). Most of these high-confidence circRNAs exhibit two annotated junctions, with 342 circRNAs displaying higher abundance compared with their host genes (Figures S1A, S1B, and Table S2). We then integrated the data into the linear mRNA counts, normalized them, and determined whether we observed changes with age (Figure S1C). After this data integration, the levels of linear and circRNAs cannot be directly compared: the quantification of circRNAs relies only on backsplicing RNA-seq reads, while the mRNA quantification uses all the reads within the transcript. We found that the total number of circRNA reads steadily increases as the fly ages in both males and females (Figures 1A and 1B). In contrast, total mRNA reads did not exhibit comparable changes with age, indicating that the age-dependent effect is specific to circRNAs. The age-dependent increase in circRNAs is evident for abundant circRNAs like cirScro, and circMbl (which hosts several circRNAs) even upon visual inspection of the RNA-seq data (Figures 1C and S1D, respectively). The increase in circRNA predominantly stemmed from higher levels of existing circRNAs, as the diversity of circRNAs types only marginally increased with age (Figures 1D and S1E). Notably, a minority of circRNAs accounted for the majority of circRNA reads: the top 25% most expressed circRNAs are over 80% of all backsplicing reads and the top three circRNAs account for more than 20% (Figures S1F and S1G). The contribution of these highly expressed circRNAs to the overall read count remained constant with age (Figures S1E and S1F top), suggesting that the age-related increase in circRNA reads is a broad phenomenon (Figure 1E).

### circRNAs outperform mRNAs as age markers

**Figure 2.**
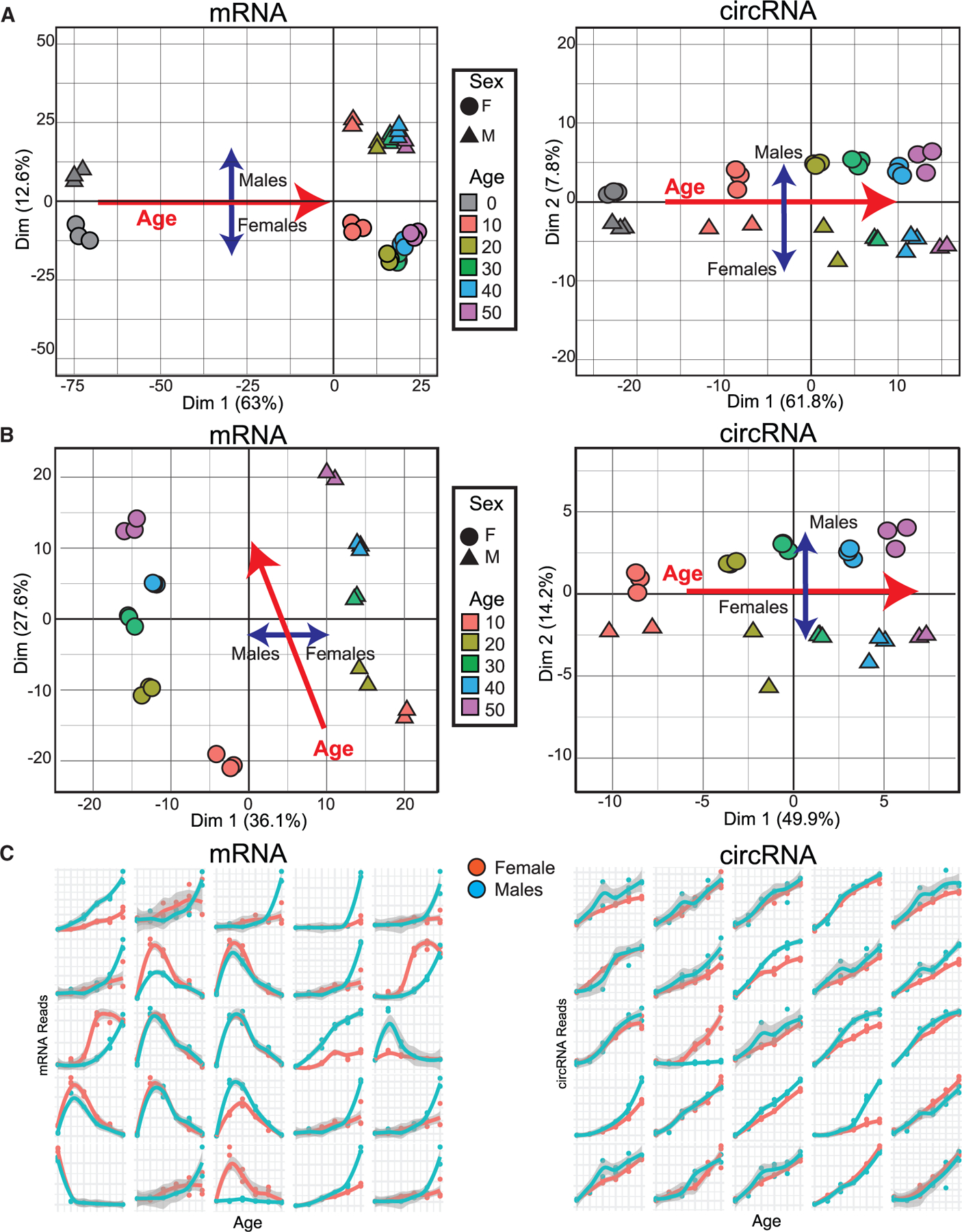
circRNAs as predictors of age (A) Left: Principal-component analysis (PCA) of the aging RNA-seq dataset of mRNA data at all available time points. Right: PCA using only circRNA data. (B) Left: PCA of the aging RNA-seq dataset of mRNA data excluding the first time point. Right: PCA using circRNA data for the same time points. Females are represented by circles, and males by triangles, with colors indicating the age time point. (C) Left: Top 25 contributors to the PCA from (B) for linear RNAs. Right: top 25 contributors to the PCA from (B) for circRNAs. Data represent the average of biological replicas for each age and sex (n = 2–3).

We utilized linear and circular RNA gene expression data to assess how efficiently it could distinguish between ages using principal-component analysis (PCA). When analyzing mRNA gene expression data, PCA effectively separated samples by sex and distinguished newly eclosed flies from the aged ones. However, the distances diminished when comparing flies older than 10 days (Figure 2A, left). In contrast, PCA based solely on circRNA data robustly differentiated samples by age, indicating that circRNAs can serve as reliable age markers (Figure 2A, right). When we performed the same analysis, excluding the first time point (“newborn” flies), sex-dependent differences predominated in mRNA expression data, overshadowing age-related differences (Figure 2B, left). Conversely, age remained the primary determinant in circRNA data (Figure 2B). We further examined the profile of the linear and circular RNAs that significantly contribute to the age-dependent PCA component (PC1 for circRNAs and PC2 for mRNAs). Notably, 24 of the top 25 circRNA contributors exhibited clear age-dependent increases and displayed similar patterns between male and female flies (see Figure 2C, right; Table S3). In contrast, mRNAs contributing to age differentiation in PCA showed variability across few time points and did not consistently exhibit similar behavior between sexes (Figure 2C, left), suggesting that mRNAs are less reliable as age markers compared with circRNAs. Together, these results demonstrate that age is the primary determinant of circRNA levels, establishing circRNAs as age markers. To determine if some of the same circRNAs displaying age-dependent accumulation could be used as age markers in another common lab strain, we determined the age-dependent profile of four circRNAs in *w*^*1118*^ male flies. Briefly, we collected heads of flies of 3, 10, 20, and 30 days of age, extracted RNA, and performed qRT-PCR. As observed in our RNA-seq data from *CantonS* flies, the tested circRNAs also displayed a very strong age-dependent accumulation in the *w*^*1118*^ strain (Figure S2A). These data show that circRNAs can be used as age markers by qRT-PCR across different fly strains.

### circRNAs exhibit three distinct expression patterns during aging

**Figure 3.**
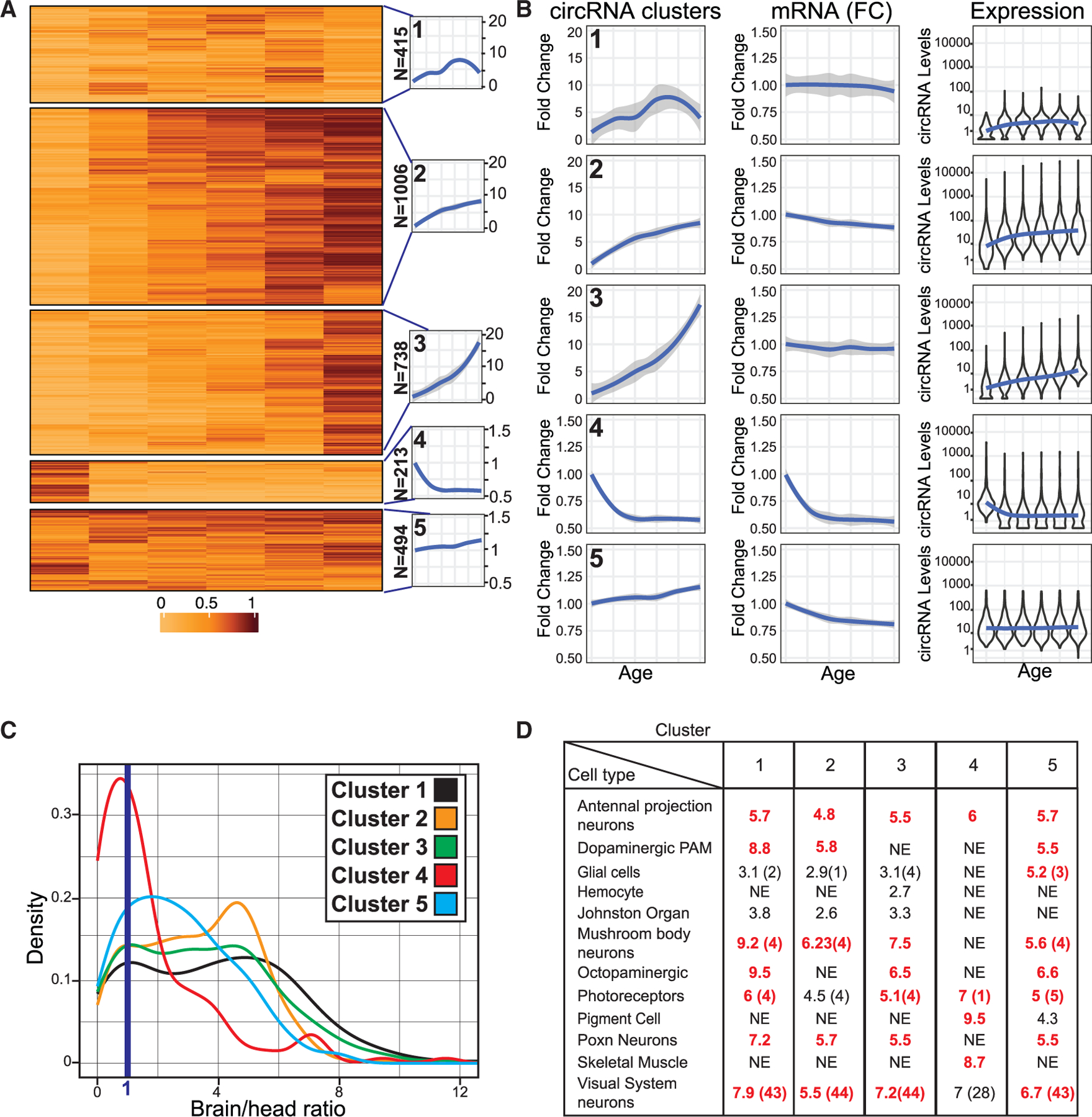
circRNAs expression follows three main patterns as fly ages (A) Heatmap of k-means clustering results of circRNA reads normalized to the first time point value for female flies, showing five identified clusters and their average expression profiles (traces on the right side). N indicates the number of circRNAs in each cluster. (B) Left: Average fold change in circRNA expression within indicated clusters compared with day 0. Center: Averaged fold change in the mRNA counterparts of the circRNAs in each cluster compared with day 0. Right: Violin plots displaying the log_10_ expression of circRNA in each cluster *per* time point, with a blue trending line indicating average values. Data are for female flies. (C) Histogram of brain enrichment values distribution for circRNAs in each cluster in females, with the blued line representing the 1:1 brain-to-head ratio threshold. (D) Summary table of cell-type enrichment analysis for genes hosting circRNAs within each female cluster using the Cell Marker Enrichment tool. Cell types significantly enriched (corrected *p* values <0.05) are listed. NE indicates no significant enrichment. Similar neuron/tissue types are summarized in one cluster, with average enrichment reported and the number of enriched cell types in brackets (see Table S6 for details).

We employed cluster analysis to determine the age-related expression patterns of specific circRNAs. To compare the profiles independently of individual circRNA expression levels, we normalized them to the first age time point (time 0, which is 2–3 days after eclosion). We identified five main clusters, with most circRNAs belonging to clusters that increase with age (clusters 1–3 in Figures 3A, S2B, and Table S4). Specifically, circRNAs in clusters 1, 2, and 3 show age-dependent accumulation, although at different rates. Clusters 4 and 5 contain circRNAs that do not increase with age: cluster 4 includes circRNAs that decrease with age, and cluster 5 circRNAs remain relatively constant as the fly ages (Figures 3A and S2B for females and males, respectively). Interestingly, the drop in cirRNA levels in cluster 4 occurs after the first time point, suggesting a development rather than aging-related change.

To determine if the age-dependent circRNA expression patterns are due to changes in the expression of the host gene, we measured the mRNA levels produced by the genes hosting the circRNAs in each cluster. Interestingly, the levels of the linear counterparts of circRNAs in clusters 1, 2, 3, and 5 remained relatively constant with age (Figures 3B and S2C). However, the linear counterparts of circRNAs in cluster 4 (which decrease with age) also decrease with age and with similar kinetics (Figures 3B and S2C), suggesting that the decrease on the levels of these circRNAs is transcriptional and that these circRNAs are short-lived, as their overall levels mirror the mRNA levels.

As shown above, circRNAs in clusters 4 and 5 deviate from the age-dependent increase observed in most circRNAs. This deviation could be due to lower production or higher degradation rates of circRNAs on these clusters. Interestingly, the steady-state levels of circRNAs in cluster 5 (invariant with age) are within the middle-high range (Figures 3B and S2C), suggesting that circRNAs in this cluster are expressed at moderate-high levels but have a higher turnover rate than those in clusters 1–3. For circRNAs in cluster 4 (which decrease with age), their levels are medium-high at the first time point (Figures 3B and S2B), and show kinetics similar to their mRNA counterparts, indicating lower production levels as flies complete development and rapid degradation.

### circRNAs with different accumulation patterns display different tissue and cellular expression

Differences in the age-dependent patterns of individual circRNA clusters may result from their distinct cellular expression patterns. Fly heads contain several tissues, including the brain, the eye, the fat body, and muscles. CircRNAs that do not increase with age might be expressed outside the brain. To test this possibility, we generated and sequenced rRNA-depleted total RNA-seq libraries from fly heads and dissected brains. We quantified mRNAs and circRNAs and assigned a brain/head ratio to each RNA and determined the brain/head expression signal distribution. Interestingly, circRNAs are more enriched in the brain than mRNAs (compare top and bottom distributions in Figure S2D; see full results in Table S4). As expected, mRNAs encoding eye rhodopsins (Rh3-6) or fat body-specific proteins (Yp1, Yp2) have low brain/head ratios (∼0.1 or 0.25, respectively). In contrast, mRNAs encoding circadian proteins *tim, Clk*, or *vri* (expressed in the brain, eye, and fat body) show ratios between 0.25 and 0.5 and *elav* mRNA (expressed in brain and eye neurons) has a ratio of 1. mRNAs encoding brain-specific proteins (like the glial marker *repo* or the dopamine enzyme *Ddc*) display ratios higher than 1. Interestingly, most circRNAs have ratios >1 (see blue line, Figure S2D), indicating predominant brain expression or share expression between the brain and other head tissue (likely the eye).

Most circRNAs in the clusters that increase with age (1–3) are strongly enriched in the brain (see Figure 3C and Table S5). This observation strongly suggests that the overall age-dependent increase predominantly occurs in neurons, specifically in the eye and brain. Conversely, circRNAs in cluster 4, which decrease with age, exhibit enriched expression outside of the brain (Figure 3C). Notably, circRNAs in cluster 5, which remains constant with age, show an intermediate level of enrichment, with a significant proportion still being enriched in the brain (Figure 3C). This intermediate enrichment implies that several circRNAs within this cluster are specific to the brain and subject to dynamic regulation. These findings suggest that these circRNAs may experience higher degradation rates in neurons or may be expressed exclusively or additionally in glial cells within the brain.

To complement these data, and considering that linear and circRNAs are typically co-expressed in the same cell types,^33^ we employed the Cell Marker Enrichment tool (DRscDB) and available single-cell data from fly heads to identify the tissues and cell types expressing genes hosting circRNAs in each cluster (see Figure 3D and Table S6 for full results). We found that genes hosting circRNAs from clusters 1–3 are highly enriched in neuronal populations, such as neurons in the visual system, dopaminergic neurons, mushroom body neurons, antennal projection neurons, and photoreceptors. Genes hosting circRNAs from cluster 4, which decrease with age, predominantly express mRNAs that are highly enriched in different cell types in the fly head, notably muscle and pigment cells. Last, genes hosting circRNAs from cluster 5 are enriched in various neuronal types, similar to clusters 1–3, but also in glial cells. In sum, our data indicate that the majority of circRNAs increase with age in the fly brain, with the circRNAs decreasing in levels with age being expressed in non-neuronal cells and regulated developmentally. Overall, our results suggest that the global age-dependent increase is specific to the brain and is closely linked to the high stability of most circRNAs in neurons.

### Divergent genomic features define circRNA cluster behavior

To further characterize the distinct age-dependent expression profiles, we compared a broad set of genomic and host gene features across the five circRNA clusters (Figure S3A). In both males and females, the average expression level of the mRNA from the host gene (linear average expression) is significantly lower for circRNAs in cluster 4 relative to those in the other four clusters (females) or in three of them (males). In addition, in females the mean exon length differed among clusters, with exons in cluster 1 generally longer than those in cluster 2 and host genes in cluster 5 displaying significantly longer exons than those in clusters 2 and 3.

We next focused on intrinsic circRNA features. In both sexes, circRNAs in cluster 3 consistently exhibited greater overall lengths (length circRNA) than those in clusters 1, 2, 4, and 5. In males, pairwise comparisons showed that cluster 3 circRNAs exceeded those in cluster 1, cluster 2, cluster 4, and cluster 5 (Figure S3A). In females, similar differences emerged. In parallel, total exonic length and exon span (the number of exons incorporated) were markedly higher in cluster 3 circRNAs in both sexes. These data indicate that circRNAs exhibiting robust age-dependent accumulation (notably in cluster 3) tend to be structurally more complex.

Analysis of complete circRNA GC content (circ gc) in females revealed that cluster 3 possessed lower GC percentages than clusters 1 and 2. In contrast, comparisons of the average exonic GC content within the circRNA (circRNA average exonic gc) in males demonstrated that circRNAs in clusters 1–4 showed significantly higher exonic GC percentages than those in cluster 5. In females, significant differences appeared between clusters 1 and 3 and between clusters 2 and 3. Finally, we compared intronic features of the host gene. In both males and females, the length of the intron immediately upstream of the circularized exons (upstream intron length) was significantly shorter in cluster 1 compared with clusters 2, 3, and 5. In addition, downstream intron length in females varied among clusters, with cluster 1 circRNAs associated with shorter downstream introns than those in clusters 2 and 3.

Together, our comparative analysis reveals that circRNAs exhibiting strong age-dependent accumulation, particularly those in cluster 3, display a distinct genomic signature: they are longer, incorporate more exons, and possess lower overall GC content relative to several other clusters. In parallel, host gene features—including shorter flanking introns in cluster 1 and higher linear mRNA expression in clusters 1–3 compared with cluster 4—indicate that both local genomic architecture and transcriptional context influence circRNA biogenesis and stability. The convergence of most of these findings in both male and female samples support a role of intrinsic sequence and structural determinants in contributing to circRNA levels during aging.

### The age-dependent increase in circRNA levels does not result from a rise in overall transcription from the host gene

**Figure 4.**
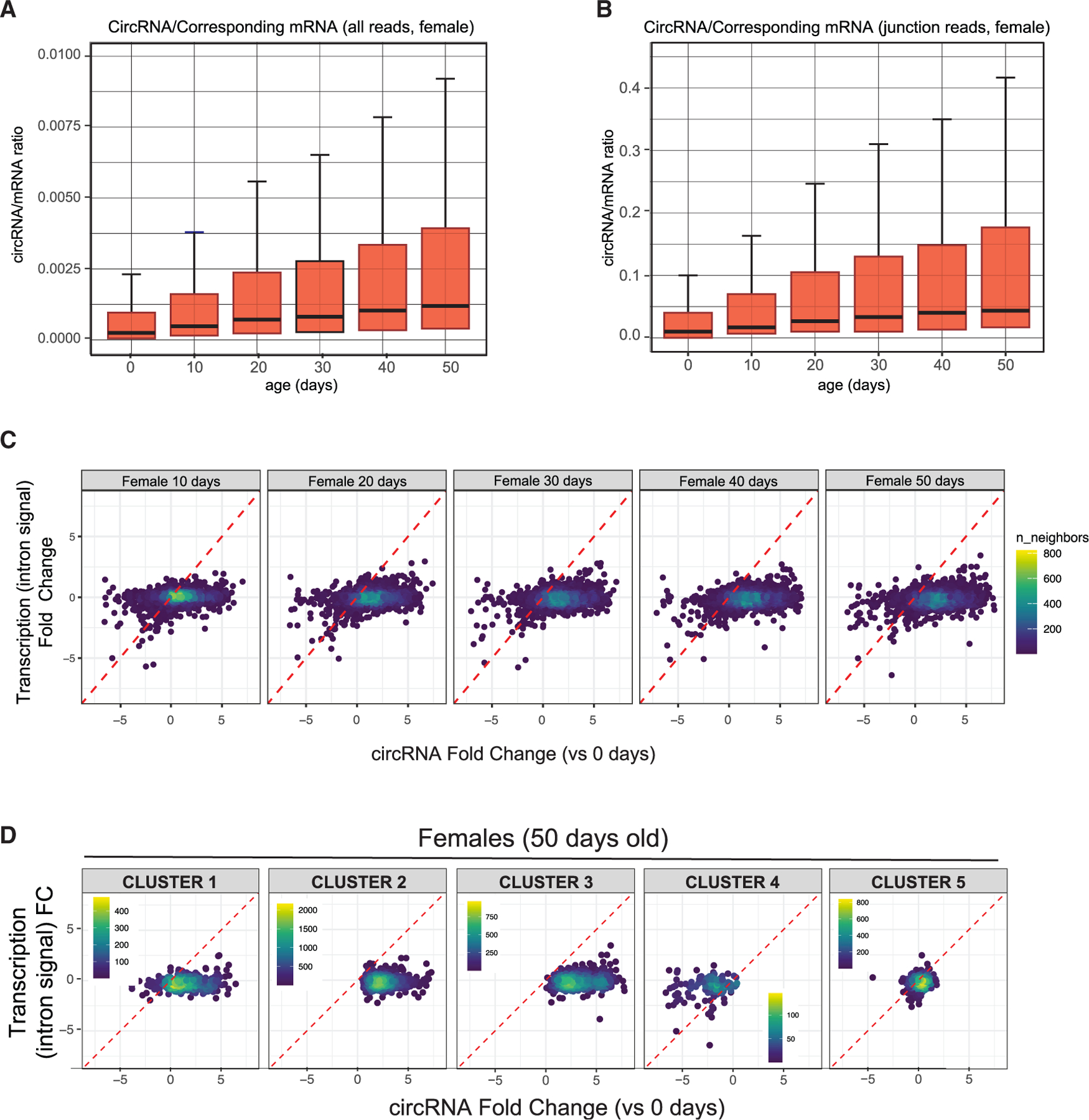
circRNAs accumulation is not due to changes in transcription (A) Boxplot of the circRNA-to-host-mRNA (circRNA/mRNA) ratio at each age in females using all mRNA-seq reads obtained by STAR alignment. (B) Boxplot of circRNA/mRNA ratios for each time point in females using only junction reads obtained in *find circ2*. (C) Scatterplots of fold changes in transcription (measured as total intron signal for the gene) and circRNA levels at days 10–50 compared with the first aging time point in females. (D) Scatterplots of fold changes in transcription (measured as total intron signal for the gene) and circRNA levels at the 50-day time point compared with day 0 for the different clusters in females.

The changes in circRNA levels as the fly ages may be due to increased biosynthesis of circRNAs and/or their accumulation due to the very low degradation rate. As illustrated in Figure 3B, most circRNAs show increased levels with age, without a corresponding increase in the linear mRNAs from the circRNA-hosting genes. Moreover, normalizing the reads of each circRNA to its linear counterpart revealed a similar increase in circRNA reads, confirming that the age-dependent rise is specific to circRNAs (Figures 4A and S3B). Importantly, we obtained similar results when normalizing the reads of each circRNA to its linear counterpart reads using either the total number of aligned reads to the mRNA (as in Figures 4A and S3B) or only the linear mRNA junction reads (the output of the circRNA detection and quantification pipeline, Figures 4B and S3C).

However, mRNA levels also are influence by degradation, which could offset age-dependent increases in transcription or circRNA production at the expense of the mRNAs. We then assessed the overall transcription levels of the genes hosting circRNAs, by measuring intronic signal. This measure, previously used by us and others as a surrogate for transcription,^57,58^ showed no changes in the total intronic signal of the genes hosting circRNAs as the fly ages (Figures 4C and S3D). This finding demonstrates that the global increase in these circRNAs is not due to higher overall transcription from the locus. This is also what we observe when we look at this relationship in clusters 1–3, which increase with age, and in cluster 5, which remains constant with age (see Figures 4D and S3E). However, the circRNAs in cluster 4, which decrease with age, display an age-dependent decrease accompanied by a reduction in the intronic signals of a fraction of the host genes, demonstrating that these circRNAs are less stable and that their decline is driven by changes in the global transcription of the entire gene (Figures 4D and S3E).

### Global age-dependent changes in circRNA levels are not attributable to splicing changes

**Figure 5.**
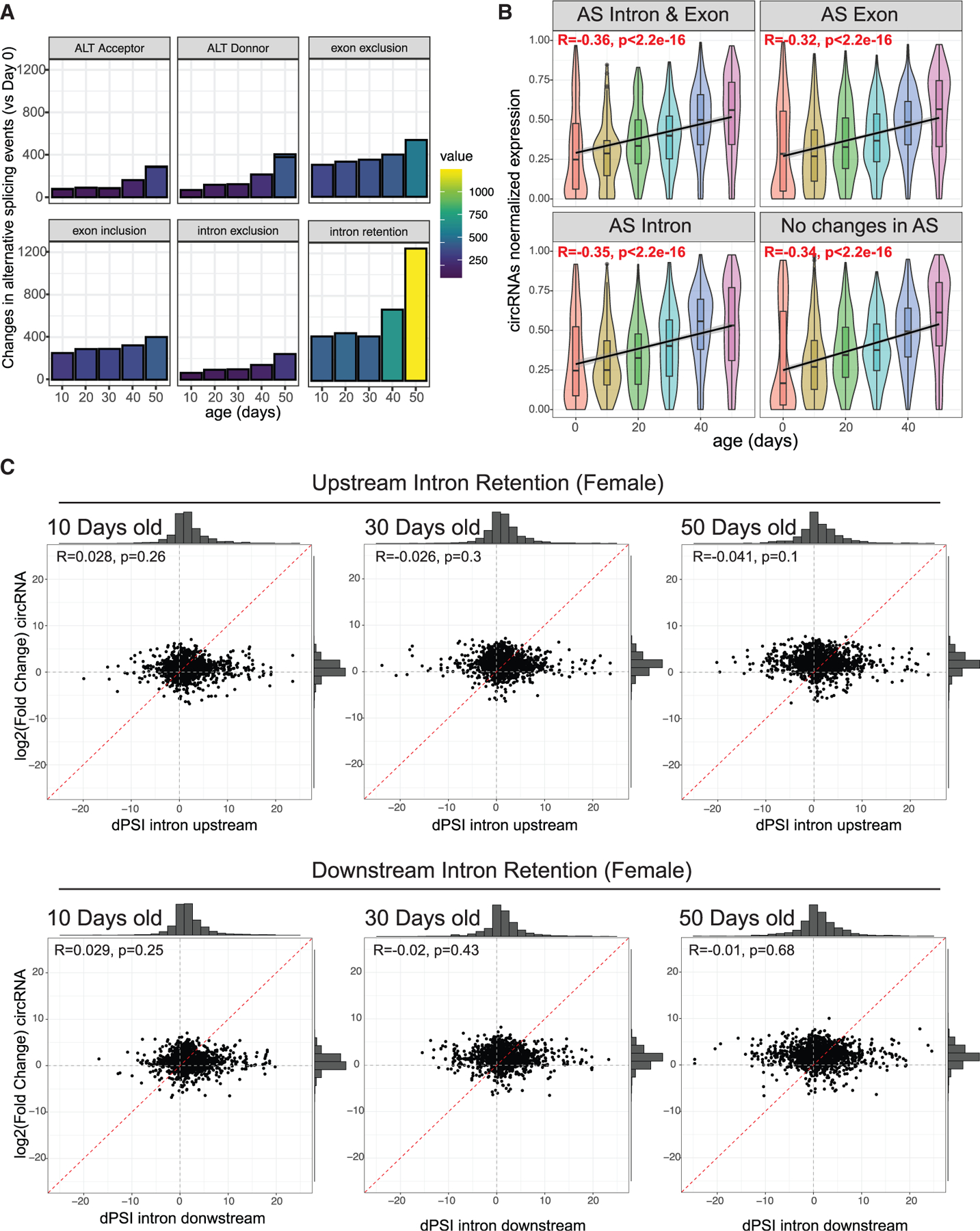
Alternative splicing changes do not primarily drive circRNA accumulation with age (A) Number of alternative splicing events differentially regulated with age in female flies. The parameters of splicing analysis tested were: alternate splice acceptor, alternate splice donor, exon exclusion, exon inclusion, intron exclusion, and intron retention. (B) Global correlation analysis of normalized circRNA expression levels with age, divided into panels based on the presence (or absence) and type of changes in alternative splicing of their host gene. (C) Correlation plots in females between circRNA fold change and vPSI for upstream (top panels) or downstream introns (bottom panels) at ages 10, 30, or 50 vs. age 0.

The findings from previous sections indicate that circRNA production via backsplicing remains relatively constant. However, it remains plausible that circRNAs could increase their synthesis without affecting the levels of linear mRNAs from the same locus, even if overall transcription levels are unchanged. This could occur if age-related increases in circRNAs depend on circRNAs produced from unprocessed pre-mRNAs. To explore this possibility, we investigated whether global splicing changes occur with age. Our analysis revealed that as the flies age, they exhibit higher levels of alternative acceptors and donors, which likely indicate increased splicing errors or reduced degradation of improperly processed transcripts (Figures 5A and S4A). We also observed an increase in the inclusion or exclusion of alternative exons. Consistent with previous reports,^59,60^ we found a substantial increase in the number of retained introns as flies age (Figures 5A and S4A, lower right panels). This trend may reflect a decline in splicing efficiency with age and/or a decrease in the degradation rates of aberrant transcripts. Notably, introns flanking circularizable exons are less efficiently spliced,^9,51^ suggesting that the age-dependent circRNA upregulation could partly result from increased intron retention. However, this explanation seems unlikely since circRNA accumulation is evident even in young flies, whereas changes in splicing only appear after 30 days, with the most significant changes at 50 days (compare Figures 1A and 1B with Figures 5A and S4A). Additionally, circRNAs originating from genes with significant changes in alternative splicing and those with no significant splicing changes exhibit similar age-related accumulation profiles, indicating no direct connection between splicing changes and overall circRNA levels (Figures 5B and S4B). To further investigate this possibility, we examined whether the changes in intron retention (measured as changes in percentage of inclusion [PSI] for upstream or downstream introns) correlate with changes in circRNA levels as a fly ages. Despite increases in both intron retention and circRNA levels with age, we found no relationship between these processes, ruling out a causal link between intron retention changes and circRNA accumulation (*p* value > 0.05 for all correlations, Figures 5C and S4C). In summary, the data presented in previous sections and here indicate that the age-dependent increase in circRNAs is not due to increased circRNA production.

### A subset of circRNAs changes their levels with temperature

**Figure 6.**
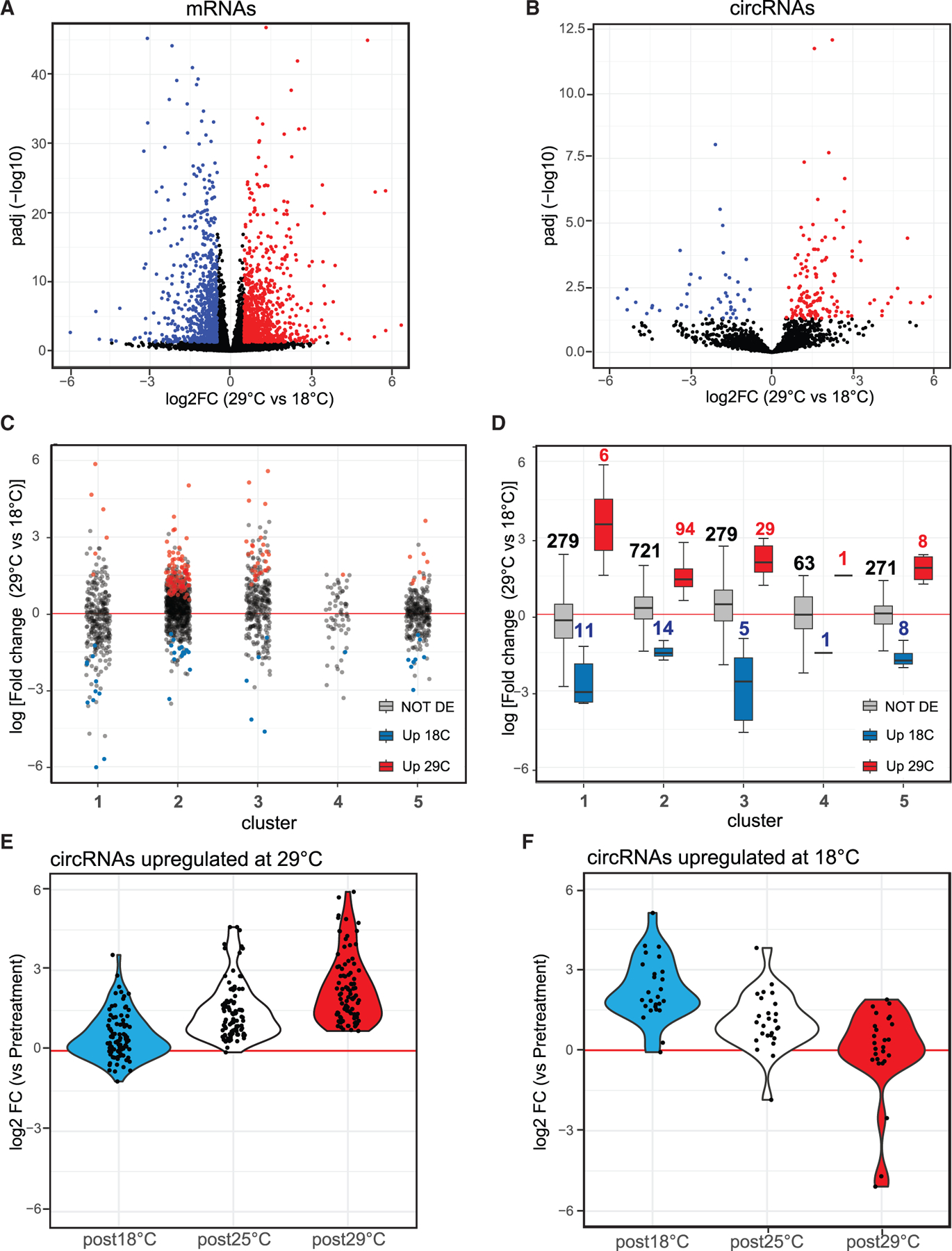
A subset of circRNAs increase in response to temperature treatment (A) Volcano plot displaying differentially expressed mRNAs in fly heads after 10 days of treatment at 29° C vs. 18° C. Red and blue points indicate mRNAs significantly upregulated at 29° C or 18° C, respectively. False discovery rate (FDR) <0.05, log2FoldChange >0.5 or < −0.5. (B) Volcano plot displaying differentially expressed circRNAs under the same conditions as in (A). (C) Dot plot illustrating the log fold change of circRNAs within the five circRNA clusters compared with the pretreatment sample. circRNAs upregulated at 18° C or 29° C are shown in blue or red, respectively. (D) Boxplots presenting the same fold-change data as in (C) but for circRNAs with no change (gray boxes), upregulated at 18° C (blue boxes), and upregulated (red boxes) at 29° C. (E) Violin plot showing the log fold change of circRNAs upregulated at 29° C across all temperatures compared to the pretreatment sample. (F) Violin plot illustrating the log fold change of circRNAs upregulated at 18° C across all temperatures compared to the pretreatment sample.

The results displayed above show that circRNAs are extremely stable. Consequently, we would expect that stimuli that alter circRNA levels to primarily increase these molecules. To test this hypothesis, we measured circRNA levels in the heads of newly eclosed flies and flies transferred to 18 or 29°C for 10 days, using flies kept at 25°C for 10 days as a control. We extracted RNA from the heads, generated and sequenced rRNA-depleted total RNA-seq libraries in triplicate, and quantified circular and linear RNAs (see gene expression data in Table S7). As expected, the total count of circRNAs increased by approximatively 40% between newly eclosed flies and those kept at 25°C for 10 days, consistent with our aging experiment (Figure S5A). Interestingly, temperature treatment slightly altered the overall number of backsplicing reads, with flies at 29°C displaying a higher number of backsplicing reads (Figure S5A). This effect on circRNA levels likely results from changes in biosynthesis due to decreased linear splicing efficiency at 29°C and alterations in RNA structure, as previously suggested,^9^ although it could also be a consequence of accelerated aging as temperature is known to alter the aging rate in *Drosophila* R.^61,62^ We identified over 1,600 differentially expressed mRNAs, with 839 upregulated and 812 downregulated (Figures 6A and S5B). Interestingly, most circRNAs were not affected by the temperature treatment; specifically, 139 circRNAs were upregulated and 46 downregulated in flies exposed to 29°C compared with those at 18°C (Figures 6B and S5B). The increase in circRNA levels is not due to accelerated aging, as only a subset of age-related circRNAs (cluster 1–3) are upregulated at 29°C, while others displayed the opposite trend (Figures 6C and 6D). Additionally, some circRNAs in cluster 5, which does not change with age, also showed increased or decreased levels at 29°C (Figures 6C and 6D). Comparing circRNA expression after a week at 29 or 18°C to pre-exposure levels revealed a general increase in circRNA levels, with only subsets of circRNAs in different clusters responding strongly to the 29° C treatment (Figure S5C). This indicates that temperature specifically increases the levels of a subset of circRNAs, distinct from the age-dependent increase.

To determine if differential circRNA expression at 29 vs. 18°C resulted from increased expression and/or degradation, we compared the levels of each differentially expressed circRNA to pretreatment levels. Only two of the 186 differentially expressed circRNAs (when comparing 29 vs. 18°C) were downregulated at one of the temperatures compared with the initial data point (Figures 6E and 6F). A few more circRNAs (four) were downregulated in both temperatures, while hundreds were upregulated (Figures S5B–S5C). These findings demonstrate that changes in circRNAs are due to increased levels at one temperature, highlighting their long stability and minimal degradation in the adult head. This contrasts with mRNAs, which exhibited both upregulated and downregulated genes at both temperatures (Figures S5B–S5D and S5E).

Interestingly, analysis of brain enrichment ratios confirmed that most circRNAs increased upon exposure to 29°C are brain-enriched (Figure S5F). Further cell enrichment analysis indicates that the genes hosting differentially expressed circRNAs are highly enriched in brain neurons, with some also highly expressed in the eye (Figure S5G). This enrichment suggests that these high-temperature-induced circRNAs are neuron-specific and highly stable.

### circRNAs display remarkably long half-lives in the fly brain and can be used as markers of environmental exposure

**Figure 7.**
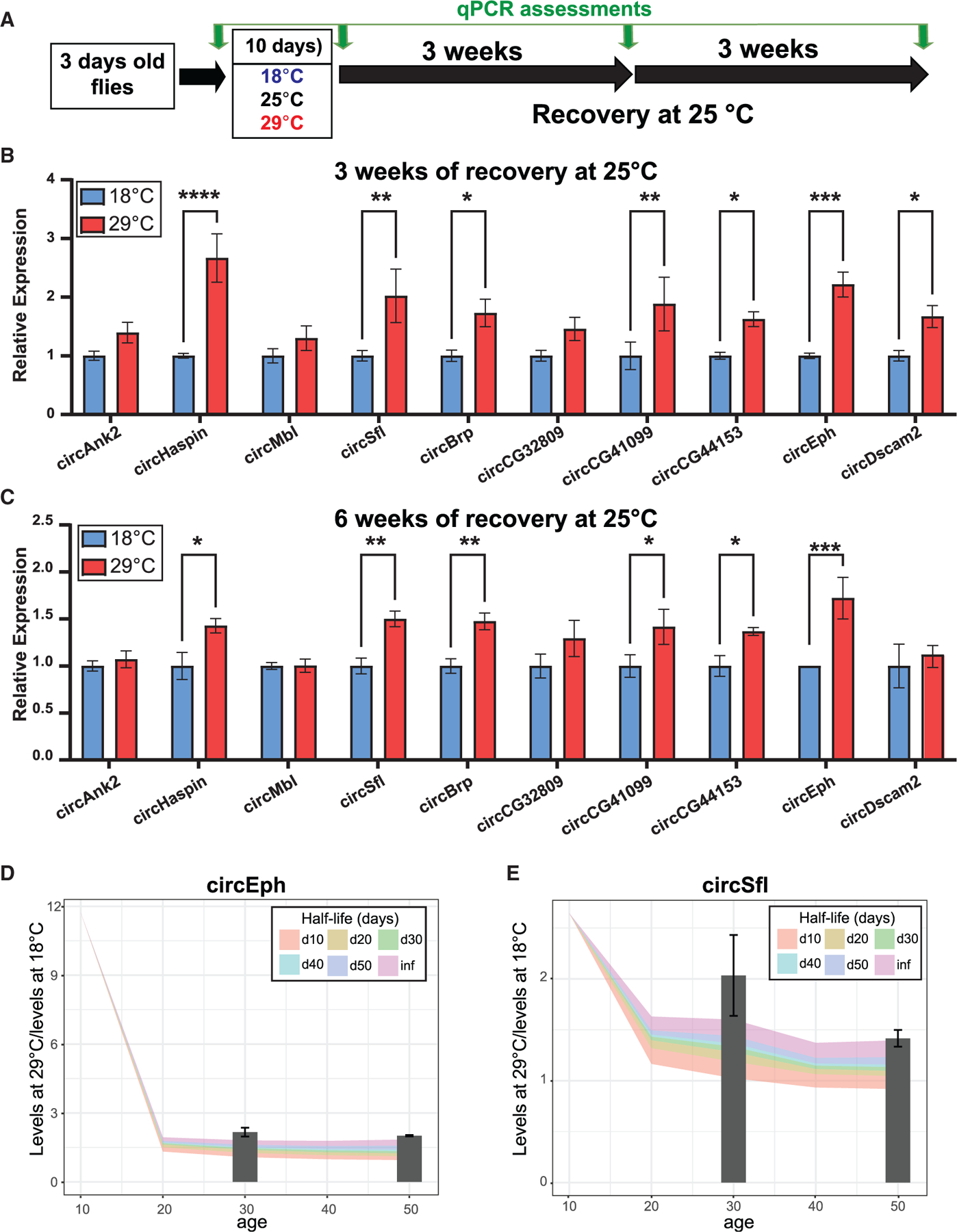
The expression of specific temperature-induced circRNAs is maintained over time (A) The experimental workflow outlines the exposure to different temperatures and their recovery times, with green arrows indicating collection times. (B) Relative expression of the indicated circRNAs in males after 3 weeks of recovery from the temperature treatment (*n* = 5). (C) Relative expression of the indicated circRNAs in males after 6 weeks of recovery from the temperature treatment (*n* = 3). qPCR results were normalized to tubulin and 28S rRNA. Means ± SEM are plotted. Significance was determined using two-way ANOVAs (*p < 0.05; **p < 0.01; ***p < 0.001; ****p < 0.0001). (D and E) The fold change between 18° C and 29° C is modeled on aging data of circEph (D) and circSfl (E). Colored bands indicated projected fold change for each half-life, while bars represent experimental RT-qPCR fold change.

Our data demonstrate that circRNAs are highly stable, and we have identified a subset of brain-enriched circRNAs that are upregulated following treatment at 29°C. These results suggest that circRNAs can serve as biomarkers for an animal’s previous exposure to specific environmental cues. To test this hypothesis, we subjected newly eclosed flies to temperatures of 18, 25, or 29°C for 10 days, followed by a return to 25°C for 3 or 6 weeks (Figure 7A). We extracted RNA from the flies before and after the temperature treatment, and again 3 and 6 weeks post-treatment. We then measured the levels of a subset of temperature-induced circRNAs using RT-qPCR. We focused on circRNAs that accumulate with age in the brain, presuming these to be the most stable ones. To identify the best markers, we included circRNAs that were both highly expressed and those that, while less abundant, exhibited a strong response to the 29°C treatment. This approach led us to select 10 circRNAs. Consistent with our RNA-seq data, all tested circRNAs showed significant upregulation following the 29°C treatment when compared with flies treated at 18°C and 25°C (Figure S6A). Three weeks after returning flies to 25°C, we observed a significant and robust upregulation of 7 out of the 10 circRNAs in flies previously exposed to 29°C, compared with those exposed to 18°C (Figure 7B), although the fold change was somehow reduced, likely because of masking due to age-dependent increases in circRNA levels. Interestingly, this effect seems to be specific to 29°C exposure, as exposure to 25°C did not result in changes in any of the four tested circRNAs when compared with 18°C 3 weeks post-exposure (Figure S6B). Notably, 6 weeks post-treatment, six of these circRNAs still displayed significantly higher levels (Figure 7C). This finding demonstrates that analyzing the expression of specific circRNAs later in life can detect transient exposure to elevated temperatures shortly after birth.

The experiment described above can be used to estimate the half-life of temperature-induced circRNAs. Briefly, we utilized the RNA-seq data to model the age-dependent accumulation in flies after day 10 and employed the RNA-seq and RT-qPCR results to calculate the amount of circRNA synthesized during the temperature treatment. We then used fold changes from our qPCR measurements at 3 and 6 weeks after returning to 25°C to estimate the half-life of the quantified circRNAs. This approach revealed that most of these circRNAs have half-lives longer than 20 days *in vivo* (Figure S6C) with some (circSfl and circEph) potentially not degrading at all (Figures 7D and 7E).

## DISCUSSION

In this study, we used genomic approaches to investigate the stability of circRNAs *in vivo* and the reasons for their age-dependent increase. RNA-seq analysis of fly heads at various ages revealed a linear increase in circRNA levels, suggesting that circRNAs are superior age markers compared with mRNAs. We attribute this accumulation to the high stability of circRNAs in neural tissue—exceeding 40–50 days in an organism with a 50- to 60-day lifespan—which also enables them to serve as markers of stress or life experiences.

Our data demonstrate that circRNA biosynthesis does not significantly change with age: we observed no global shifts in mRNAs from circRNA-producing loci, no correlation between circRNA levels and transcriptional output (assessed by intronic reads^57,58^), and no link to splicing changes (e.g., intron retention). This contrasts with suggestions that circRNA increases could reflect RNA processing alterations during aging.^60^ While we cannot fully exclude minor production-rate changes, high circRNA stability remains essential to explain their accumulation. Future studies using pulse-chase labeling or chromatin/nuclear RNA measurements could refine these findings. It is also possible that circRNA stability itself might increase with age, though our data suggest circRNA levels plateau rather than accelerate. Moreover, circRNAs might track biological as well as chronological age, since circSfl is regulated by the insulin pathway; exploring long- or short-lived mutants could clarify this.

Interestingly, not all circRNAs increase with age. A subgroup of circRNAs decreases as flies age, mirroring effects on their linear counterparts and overall transcriptional output. These circRNAs are expressed outside the brain, likely in dividing cells and tissues, and appear to degrade rapidly in adulthood. Conversely, the other four clusters likely contain circRNAs expressed predominantly in neurons, with some circRNAs in cluster 5 expressed also in glial cells. While circRNAs in clusters 1–3 accumulate due to high stability, circRNAs in cluster 5 maintain constant levels with age, suggesting higher degradation rates. These differences may be due to expression in glia and/or dynamic degradation in response to stimuli like electrical activity, which has been shown to promote circRNA degradation in mammals,^32^ warranting further study.

We found that temperature treatment altered the expression of ∼180 circRNAs, mostly upregulated, consistent with prior reports of global circRNA increases at 29°C^9^; however, the sequencing depth was insufficient to identify specific circRNAs. Our deeper sequencing now attributes this mainly to a few highly abundant circRNAs. We hypothesize these changes arise from decreased linear splicing efficiency or alterations in RNA editing/structure.^8,9,33,51^ Interestingly, we predominantly observed increases in circRNA levels, identifying only six circRNAs that were downregulated following any of the treatments, in contrast to numerous mRNAs. One might argue that the increase in circRNA levels is due to accelerated aging at 29°C. However, this is unlikely, as there is little correlation between the circRNAs increased by temperature treatment and those that accumulate with age. Indeed, many circRNAs that exhibit dramatic age-dependent accumulation are not affected by temperature treatment. Indeed, it would be interesting to investigate whether any of these temperature-induced circRNAs play any role in cellular or physiological responses to adaptation to 29°C.

Additionally, our data reveal that circRNAs can indicate previous exposure to 29°C, even if this occurred 6 weeks prior. This is an exciting development and is linked to the extraordinary stability of these molecules. We estimate that these half-lives of some of these circRNAs position them as some of the most stable molecules in a living organism, surpassing even the stability of some of the most stable proteins. These half-lives exceed those of very stable RNAs and are significantly longer than previously suggested for circRNAs based on cell culture studies. It will be very interesting to determine how many circRNAs and for how long after flies are transferred to 25°C remain upregulated and can serve as markers of exposure to higher temperature. Furthermore, it would be interesting to examine whether other environmental conditions or stresses (such as lightening conditions, exposure to toxins, antibiotics, starvation, or desiccation) induce specific circRNAs patterns that persist over time. If this is the case, circRNAs could reveal the integrated transcriptome of a fly population and the types and durations of environmental stresses endured. This could be particularly relevant in the context of climate change, where insects and other animals face unpredictable acute and long-term exposure to unusual environmental and ecological conditions. Additionally, this new application of circRNAs as stress sensors could be expanded to mammals to reveal exposure to toxins and potentially uncover underlying diseases.

### Limitations of the study

We acknowledge that our study has several limitations. First, our approach relies on indirect estimates of circRNA half-lives rather than direct measurements (e.g., through pulse-chase labeling). Thus, we cannot entirely exclude the possibility that subtle, age-related changes in circRNA biogenesis also contribute to their accumulation. In addition, our temperature exposure experiments were conducted on bulk fly head samples, which limits our ability to resolve cell-type-specific responses.

## RESOURCE AVAILABILITY

### Lead contact

Further information and request of reagents should be addressed to Prof. Sebastian Kadener (skadener@brandeis.edu).

### Materials availability

All unique/stable reagents generated in this study are available from the lead contact with a completed material transfer agreement.

### Data and code availability

- All next-generation sequencing data have been deposited at the GEO repository under the accession numbers GSE261524 and GSE261460 and are publicly available as of the date of publication.
- The custom code generated in this study has been deposited in Zenodo: https://doi.org/10.5281/zenodo.14976887.
- This study did not generate new unique reagents.
- Any additional information is available from the lead contact upon request.

## STAR★METHODS

### EXPERIMENTAL MODEL AND STUDY PARTICIPANT DETAILS

All the experiments were done utilizing CantonS (Stock 6366, Bloomington *Drosophila* Stock Center, Indiana, USA) or *w1118* (Stock 5905, Bloomington *Drosophila* Stock Center, Indiana, USA) as indicated. The sex and age of the utilized flies depends on the experiments and it is indicated below and in the figure legends and was 2–3days, 10, 20, 30, 40 and 50 days of age. The temperature treatment experiments were performed in 3-day old male flies. In all experiments we utilized fly heads except for calculating brain/head ratios.

### METHOD DETAILS

#### Sample collection and RNA extraction

##### Aging RNA-seq experiments

We collected age-matched CantonS (Bloomington *Drosophila* Stock Center, Indiana, USA) flies and allowed them to mate for three days before separating males and females for aging. We designated flies collected 2–3 days after eclosion flies as time 0 for simplicity. All flies were maintained at 25° C under a 12 h light and 12 h dark cycle. Samples were flash frozen in liquid nitrogen at 0, 10, 20, 30, 40, and 50 days old. Fly heads (∼100 per sample) were collected using brass sieves stored at −80C. We collected three biological replicas *per* condition.

##### qRT-PCR aging profiles from w1118 flies

We collected age-matched *w1118* (Bloomington *Drosophila* Stock Center, Indiana, USA) male flies and allowed them to mate with females before separating for aging. We designated flies collected 2–3 days after eclosion flies as time 0 for simplicity. All flies were maintained at 25°C under a 12-h light and 12-h dark cycle. Samples were flash frozen in liquid nitrogen at 0, 10, 20 and 30 days old. Fly heads (∼75 per sample) were collected using brass sieves stored at −80C. We collected three biological replicas *per* condition.

##### Exposure to temperatures and recovery experiments

We reared wild-type CantonS flies at 25°C in bottles and, after two days of mating, separated into vials containing ten males or ten females. Several vials were pooled together for a final number of ∼30 males or females *per* replica. A day was allowed for their recovery from the CO_2_ exposure before placing the flies at 18, 25, or 29°C for a ten-day period. After this period, we transferred the flies back to 25°C for three or six weeks for recovery. A pre-condition sample was collected right before placing the flies in their respective conditions, a post-condition following the ten days of exposure, and the recovery samples after the indicated times. Three to five biological replicas *per* condition were collected. RNA from the fly heads was extracted using TRIzol reagent (SIGMA) and treated with DNAseI (NEB) following the manufacturers’ protocols.

##### Generation of total RNA libraries

We extracted total RNA from fly heads or brains using TRIzol (SIGMA) reagent following the manufacturer’s instructions and treated it with DNAse I (NEB). We performed ribodepletions following a protocol based on.^65^ Briefly, 1 mg of RNA was denatured at 95 C with rRNA DNA oligos in 200mM NaCl, 100mM Tris-HCl pH 7.5, and 50 mM MgCl2 and ribodepleted using Hybridase thermostable RNaseH (Epicentre, H39500). Afterward, RNA was extracted using RNAClean XP (Beckman Coulter, A63987), DNAse treated using TurboDnase (Ambion, AM1907), and re-purified using the RNA Cleanup XP beads. We prepared total RNA libraries using the NEXTFLEX Rapid Directional RNA-Seq Kit 2.0 (PerkinElmer) as recommended by the manufacturer. Samples were sequenced by Novogene (Novogene Corporation Inc 8801 Folsom BLVD, Suite 290, Sacramento, CA) with HiSeq-4000.

##### Real-time RT-qPCR analysis

We synthesized cDNA from the extracted RNA using iScript and random primers (Bio-Rad) and diluted 1:40 before performing the quantitative real-time PCR using SYBR green (Bio-Rad) in a CFX384 C1000 Thermal Cycler (Bio-Rad). We used the following primers for amplifying each circRNA: circMbl (5′-AGGACACCGAATGCAAGTTC-3′ and 5′-AAACGCAGCTGTTAATTTTTG-3′), circAnk2 (5′-AACAG CAGCAGTCCCAGTCT-3′ and 5′-TCATCATCACCACCACCAAC-3′), circHaspin (5′-GAACTTTTCCAGGCAACAGG-3′ and 5′-TCT CCAAAAAGTTCCGGATG-3′), circSfl (ATGTCGATACGGGCGTGTTT and CCAGACTGTCCACTCGCAAT), circBrp (GAGCCGTGCGT TTGATATCA and TTTGTGGTTGTTGTCAGGCG), circCG32809 (ACCCCATGCACCAGAGTAAA and GAGAGCGAACGACCCCAT), circCG41099 (AGACGTCGTTTCTCTTTGCA and ACTTTGCACCGCCAAGATTT), circCG44153 (GTCGGGCGTTATGGAAAGAC and CTTGCACAGGGGTCCATGA), circEph (GATCGTCTCTGGATTGATTCT and ACGTACAGGAATTTTACCGCC), circCamKI (GGGGTC CTACACAGAAAAGGA and CCGCGTTCCATTTAGACATT), circScro (GAATCGTATCGCAAACTGGAA and GCGATGACATGCGTAAC AAT), circJupiter (CACCGAGAACCTCAAGATGA and TCTCTCTGTTGAATGGATGCA) and circDscam2 (TACGCCGCAAATTT CATGGA and CTTCGAACGGTTCACTGCAG). The PCR mixture was subjected to 95°C for 3 min, followed by 40 cycles of 95°C for 10 s, 55°C for 10 s, and 72°C for 30 s, followed by a melting curve analysis. We plotted fluorescence intensities against the number of cycles using an algorithm provided by the manufacturer (CFX Maestro Software, Bio-Rad). The results were normalized against 28S (5′-ATTCAGGTTCATCGGGCTTA-3′ and 5′-CCGTGAGGGAAAGTTGAAAA 3′) and/or tub (5′-TGCTCACGAAAAGCTCTCCT-3′ and 5′-CACACACGCACTATCAGCAA-3′) levels as indicated. For the life experience experiments data was plotted and analyzed using GraphPad Prism. two-way ANOVA followed by Tukey’s multiple comparisons tests were performed using GraphPad Prism version 10.1.2 for Windows (GraphPad Software, Boston, Massachusetts USA, www.graphpad.com). For the analysis of age-dependent circRNA patterns in w^1118^ flies we performed a linear regression of expression versus time.

#### Bioinformatic analysis

##### Linear RNA alignment and quantification

We aligned raw reads to the *Drosophila melanogaster* genome (dm6) using STAR.^63^ We quantified mapped reads using *featureCounts*.^66^ Using a custom R script along an intronic region reference, we extracted intronic reads from the *featureCounts* output. Two male samples from were considered outliers after PCA analysis (10 days replica 2 and 20 days replica 3) and were not utilized for any of the displayed analysis.

##### circRNA detection and quantification

We detected circRNAs by searching for head-to-tail splice junctions in unaligned reads using *find_circ2* as previously described.^6^ This analysis provided the number of annotated junctions and the circRNA/linear mRNA ratio for each sample which we used for Figure S1A. We normalized circRNA reads using size factors computed by DESeq2,^64^ along with all mapped reads. We filtered out circRNAs shorter than <50 nucleotides and removed rows that did not have an average of 1 read at every time point or at least one sample with ten reads. We calculated the fold change between each age and newly hatched flies (day 0) using a generalized linear model and a negative binomial distribution.

##### General circRNA characterization

To determine the changes of circRNA across age, we summed the normalized reads obtained from DESeq2 for each replicate. We calculated the regression equation, R^2^, and *p*-value using the *lm*() function in R.

##### Quantification of circRNA isoforms

We quantified the number of different circRNA isoforms by counting circRNAs with reads above 0 in the normalized read matrix obtained from DESeq2. We normalized the reads after filtering out circRNAs expressed at very low levels or likely artifacts.

##### Quantification of circRNA reads in quantiles with age

We assigned quantiles using the *quantile* function in R and then computed the total/average number of reads within them. We calculated quantiles separately for each replicate and then averaged them.

##### PCA analysis of mRNA and circRNA data

We performed PCA using the *prcomp*() function in R and plotted the data using the *fviz_pca_ind*() function from the *factoextra* package.

##### Clustering

We performed hierarchical clustering using foldchange values and consensus k-means clustering from 500 repetitions. We chose 5 clusters based on within-cluster sum of squares (WSS), cluster silhouette, and the similarity of clusters between sexes calculated by the Rand index. Although the WSS suggested 6 clusters for females and 5 for males, and the silhouette suggested 5 clusters for females and 6 for males, we selected 5 clusters due to the Rand index indicating the greatest increase in cluster similarity at this number.

##### Differential expression analysis

We used DESeq2 for normalization and differential expression analysis. We normalized circRNA and mRNA reads together. We identified significantly up- and down-regulated transcripts using the following parameters: log2FoldChange >0.5 or < − 0.5 and padj <0.05.

##### Computing circRNA/mRNA ratios

We calculated circRNA/mRNA ratios using two methods. First, in the “all reads” method, we determined the circRNA/host mRNA ratio by comparing circRNA reads to linear reads obtained from STAR. This was achieved by dividing circRNA reads by host gene reads after DESeq2 normalization. We identified the host gene of circRNAs using annotations assigned by find_circ. CircRNAs without identifiable host genes (classified as “intergenic”, “ambiguous,” or “no_single_host”) were excluded from this analysis. Our second method, referred to as “junction reads” in the text and figures, utilized a ratio calculated automatically by find_circ. This method involved dividing the circRNA reads by the linear reads of the host transcripts, using junction reads to quantify mRNA levels. If no linear RNA was detected, we assigned a value of 1 to the linear reads, resulting in a ratio equal to the number of circRNA reads.

##### Genomic feature extraction and analysis of circRNAs

We used the *Drosophila melanogaster* dm6 genome assembly was used for circRNA feature analysis. Ambiguous circRNAs, those from intergenic regions, those without single host genes, and circRNAs shorter than 20 nucleotides were removed during initial filtering. GC content was calculated for each circRNA using the BSgenome.Dmelanogaster.UCSC.dm6 package. Features from host genes were extracted using gene annotations from the dm6 GTF file to characterize the genomic context of each circRNA. The total number of exons, mean exon length, total exon length, and exon GC content were calculated for each host gene. Flanking introns were identified by analyzing the genomic coordinates relative to the circRNA boundaries, with strand orientation being taken into consideration. The lengths and GC content of these flanking introns were determined. Statistical differences between circRNA clusters were tested using Kruskal-Wallis tests. For features showing significant differences (*p* < 0.05), pairwise comparisons between clusters were performed using Dunn’s test. All analyses were conducted using R (version 4.1.0) with the following key packages: GenomicFeatures, Biostrings, rtracklayer, and tidyverse. A custom R script was developed to summarize pairwise comparisons of key genomic features associated with circRNA clusters. Visualizations were generated with ggplot2 (R version 4.1.0). In the heatmap, each tile represents the *Z* score for a pairwise comparison. Statistically significant comparisons were annotated with asterisks (*** for *p* < 0.001, ** for *p* < 0.01, and * for *p* < 0.05). To emphasize sex-specific differences, the plot was faceted by sex.

##### Brain/head ratio

To evaluate circRNA enrichment in the brain, we calculated the ratio between circRNA levels in brain and head tissues. We analyzed total RNA sequencing data from the heads and brains of a wild type strain (CS strain). We then calculated the ratio between the normalized reads for each circRNA in each tissue. For circRNAs with zero reads in head data, we assigned a value of one, resulting in a ratio equal to the number of normalized reads in the brain sample.

##### Cell enrichment analysis

For each cluster, we utilize *Drosophila* mRNA gene expression single-cell head data from the fly atlas^67^ and determined the enrichment and statistical significance using the Cell Marker Enrichment tool (DRscDB, https://www.flyrnai.org/tools/single_cell/web/enrichment). We included cell types identified as enriched and with corrected *p*-values <0.05 in the table. We summarized cell clusters belonging to similar neuron/tissue types in a single term and reported the average enrichment. For cell/tissue types with multiple enriched cell clusters, we indicated the number of cell cluster types in brackets.

##### Intronic reads quantification and analysis

We aligned reads with STAR-aligner^63^ to the Drosophila genome and transcriptome version dm6. We extracted rand counted intronic reads using the featureCounts function in R with an intronic region reference. We retained only signals from introns flanking circular RNAs with annotated junctions for further analysis. We incorporated these reads into the circular and linear reads and normalized them using DESeq2. We computed fold change between each age group and newly hatched flies (day 0) using a generalized linear model and a negative binomial distribution.

##### Identification of circRNAs differentially expressed by temperature treatment

For this analysis we filtered expressed circRNAs by requiring that each circRNA have at least one condition where all replicas had a count greater than 3. To identify differentially expressed (DE) circRNA post-treatment, we applied similar parameters than detailed above (log_2_FoldChange > 0.5 or < − 0.5 and padj < 0.05). Additionally, to identify DE circRNAs between the 18 and 29°C treatments, we required that these circRNAs also be differentially expressed when compared to the pre-treatment condition.

##### Alternative splicing analysis

We quantified the percentage of splicing inclusion (PSI) using Vast-Tools as previously described^68^. We then calculated Delta PSI from the mean of each condition. To determine splicing events that change between each age group and newly hatched flies (day 0), we selected events with a minimum of 15% difference in mean PSI and no overlap between replicas.

##### Half-life analysis

We calculated the fold change between 18°C and 29°C for circRNAs immediately following the temperature treatment (day 10) by averaging the fold change derived from differential expression analysis of RNA-seq data and RT-qPCR data. To model age-related changes in circRNA levels, we multiplied the fold change by the average reads of 10-day old male flies from the aging dataset. We then modeled the expected levels of circRNA as a simple exponential decay, assuming the additional circRNA produced during the temperature treatment would not affect circRNA levels for that gene further. We calculated expected circRNA levels for half-lives of 10, 20, 30, 40 and 50 days, as well as an infinite half-life (no decay). We then compared the expected fold change between 18°C and 29°C for 30-day old and 50-day old flies to experimental qPCR results at 3 weeks (aged 31 days) and 6 weeks (aged 52 days) after treatment.

### QUANTIFICATION AND STATISTICAL ANALYSIS

All the experiments and analysis were tested for statistical significance utilizing the tests, procedures and parameters indicated in the method section and figure legends. Significance was defined as corrected p < 0.05 for the tested as indicated. N = 3 or more refers to experimental replicates. All the experimental replicates are biological replicas.

## Supplementary Material

1

2

3

4

5

6

## Figures and Tables

**Table:** KEY RESOURCES TABLE

REAGENT or RESOURCE	SOURCE	IDENTIFIER
Deposited data		
RNAseq data from different ages (fly heads)	This study	GSE261460 (Gene Expression Omnibus, GEO)
RNAseq data from temperature treatment (fly heads)	This study	GSE261524 (Gene Expression Omnibus, GEO)
Experimental models: Organisms/strains		
*D. melanogaster*: *w*^1118^	Bloomington Drosophila stock center	BDSC: 5905 FlyBase:FBal0018186
*D. melanogaster*: Canton S	Bloomington Drosophila stock center	BDSC: 6366
Oligonucleotides		
Oligos for qPCR	This paper	See STAR Material and methods section
Software and algorithms		
STAR	Dobin et al., 2013^63^	https://github.com/alexdobin/STAR
DEseq2	Love et al., 2014^64^	https://bioconductor.org/packages/release/bioc/html/DESeq2.html
Find_circ	Memczak et al., 2013^6^	https://github.com/marvin-jens/find_circ
ciRcus	N/A	https://github.com/BIMSBbioinfo/ciRcus
Code generated in this study	This study	ZENODO. https://doi.org/10.5281/zenodo.14976887

## Highlights

- CircRNAs accumulate linearly with age in the fly brain
- Their exceptional stability outperforms mRNAs as age markers
- Specific circRNAs reveal prior 29°C exposure
- circRNAs serve as reliable indicators of stress and experience
