# Supplementary material for: Circular RNAs exhibit exceptional stability in the aging brain and serve as reliable age and experience indicators
Source: Cell Rep. Author manuscript; Available in PMC 2025 May 26. (PMC12105716; doi:10.1016/j.celrep.2025.115485)
Supplement: 1 [file NIHMS2076717-supplement-1.pdf]

Cell Reports, Volume *44*

Supplemental information

Circular RNAs exhibit exceptional stability in the aging brain and serve as reliable age and experience indicators

Ken Kirio, Ines Lucia Patop, Ane Martin Anduaga, Jenna Harris, Nagarjuna Pamudurti, The Nandar Su, Claire Martel, and Sebastian Kadener

Document S1: Figures S1-S7, Tables S1 and S3

Figure S1. circRNAs increase as the fly ages

Figure S2. circRNA expression follows three main patterns as fly ages.

Figure S3 circRNA expression follows three main patterns as fly ages.

Figure S4. Alternative splicing changes do not primarily drive circRNA accumulation with age.

Figure S5. A subset of circRNAs increase their levels in response to temperature treatment.

Figure S6. circRNAs can be used as life experience markers.

Table S1. Statistic of alignments for aging RNAseq samples.

Table S3. Main age determinants for circRNAs. PC1 from the PCA plotted in Figure 2B.

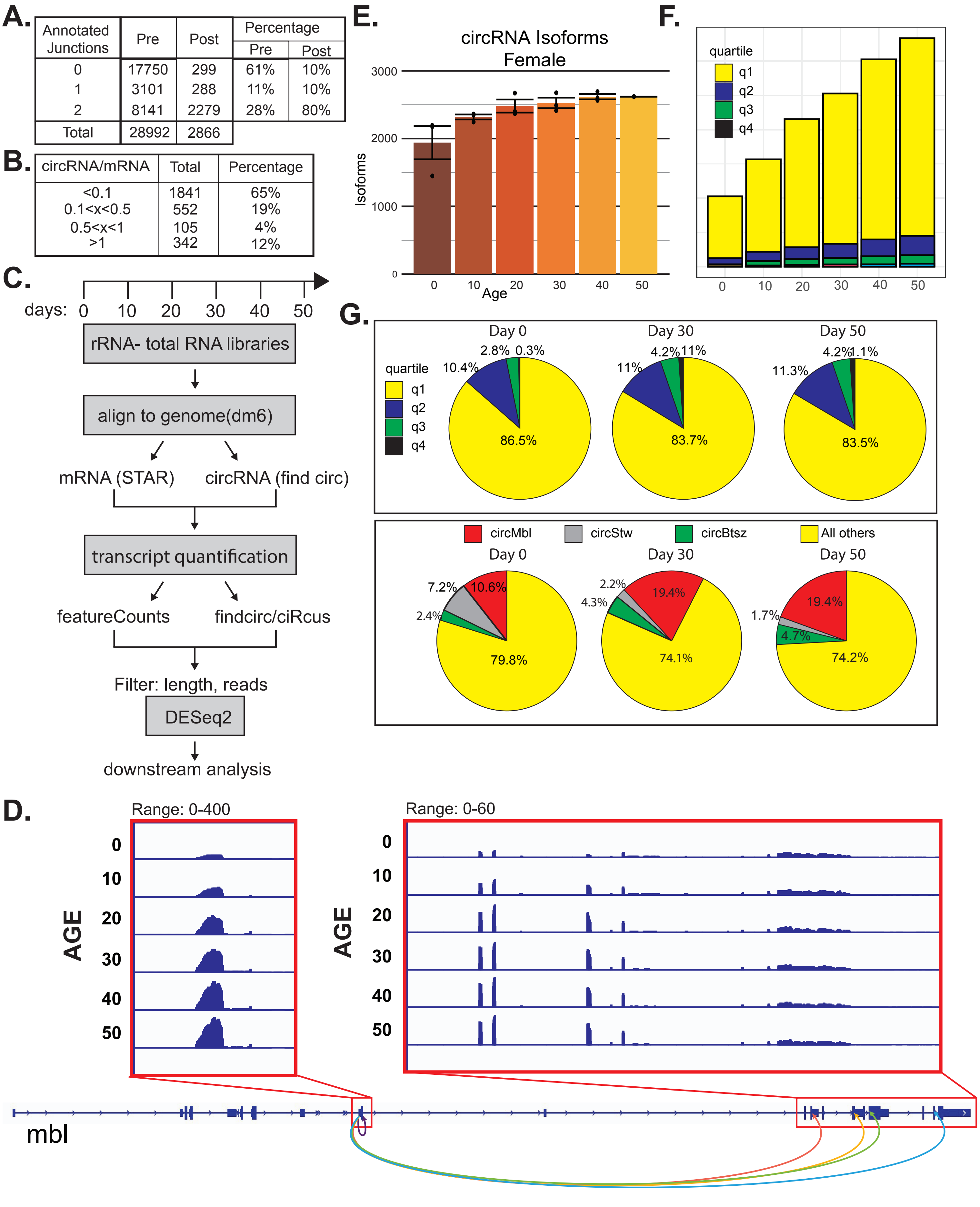

Annotated Junctions	Pre	Post	Percentage	
			Pre	Post
0	17750	299	61%	10%
1	3101	288	11%	10%
2	8141	2279	28%	80%
Total	28992	2866		

circRNA/mRNA	Total	Percentage
<0.1	1841	65%
0.1<x<0.5	552	19%
0.5<x<1	105	4%
>1	342	12%

Figure S1.

Figure S1. circRNAs increase as the fly ages. A. Top: Table showing the number and percentage of circRNAs and their types by the number of annotated junctions before and after filtering. Bottom: Number of circRNAs passing different expression thresholds, calculated using the circRNA/mRNA ratio from the *findcirc2* pipeline and the highest ratio among all timepoints and conditions. **B.** Schematic representation of the bioinformatic analysis workflow. **C.** IGV snapshot in the *mbl* gene indicating the position of the five most abundant circMbl isoforms. **D.** Number of different circRNA isoforms in females as the fly ages. Data are presented as means ± SEM. **E.** Cumulative graphs showing the number of backsplicing reads for each expression quartile as fly ages. **F.** Top: Pie charts showing the proportion of backsplicing reads originating from each expression quantile at the indicated ages. Bottom: Pie charts showing the proportion of backsplicing reads originating from the three most expressed circRNAs at the indicated ages. **Related to Figure 1.**

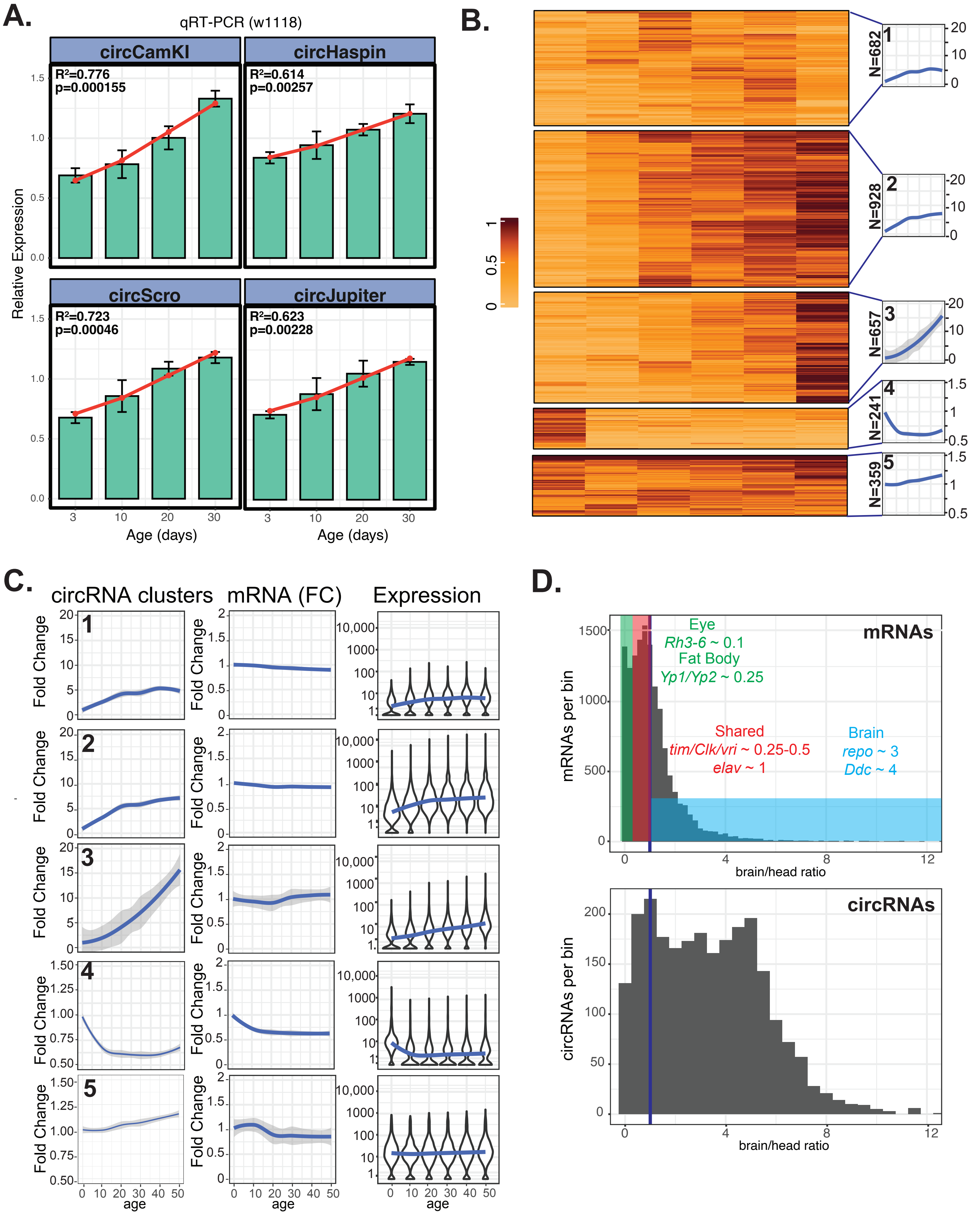
A.
qRT-PCR (w1118)
circCamKI
R²=0.776
p=0.000155
circHaspin
R²=0.614
p=0.00257
circScro
R²=0.723
p=0.00046
circJupiter
R²=0.623
p=0.00228
Relative Expression
Age (days)
B.
N=682
N=928
N=657
N=241
N=359
C.
circRNA clusters
mRNA (FC)
Expression
Fold Change
age
D.
mRNAs
Eye
Rh3-6 ~ 0.1
Fat Body
Yp1/Yp2 ~ 0.25
Shared
tim/Clk/vri ~ 0.25-0.5
elav ~ 1
Brain
repo ~ 3
Ddc ~ 4
mRNAs per bin
brain/head ratio
circRNAs
circRNAs per bin
brain/head ratio

Figure S2.

Figure S2. circRNA expression follows three main patterns as fly ages. A. qRT PCR of w1118 flies at the indicated timepoints. Graph indicates average and error refers to standard error of the mean (N=3 for each timepoint). For each profile a linear regression was calculated, plotted and the R^2 and pvalues reported. **B.** Heatmap of k-means clustering results of circRNA reads normalized to the first timepoint value for male flies, showing five identified clusters and their average expression profiles (traces on the right side). N indicates the number of circRNAs in each cluster. **C.** Left: Average fold change in circRNA expression within indicated clusters compared to day 0. Center: Averaged fold change in the mRNA counterparts of the circRNAs in each cluster compared to day 0. Right: Violin plots displaying the log10 expression of circRNA in each cluster *per* timepoint, with a blue trending line indicating average values. Data are for male flies. C. Histogram of brain enrichment values distribution for mRNAs (top) and circRNAs (bottom), with the blue line representing the 1:1 brain-to-head ratio threshold. **D.** Histogram of brain enrichment values distribution for circRNAs in each cluster in males, with the blue line representing the 1:1 brain-to-head ratio threshold. **Related to Figure 3.**

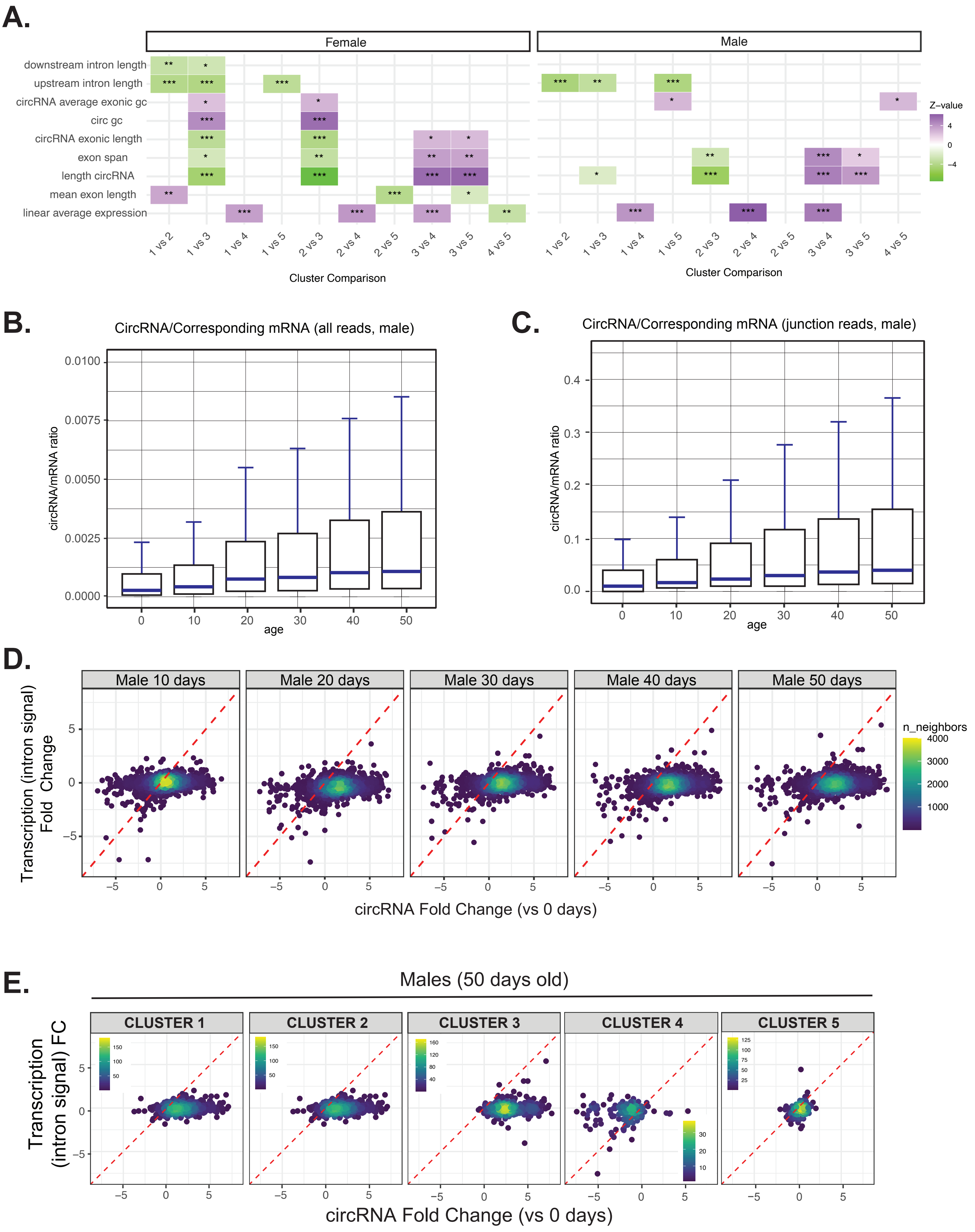
A.
Female
Male
downstream intron length
upstream intron length
circRNA average exonic gc
circ gc
circRNA exonic length
exon span
length circRNA
mean exon length
linear average expression
Z−value
1 vs 2
1 vs 3
1 vs 4
1 vs 5
2 vs 3
2 vs 4
2 vs 5
3 vs 4
3 vs 5
4 vs 5
Cluster Comparison
B.
CircRNA/Corresponding mRNA (all reads, male)
circRNA/mRNA ratio
age
C.
CircRNA/Corresponding mRNA (junction reads, male)
circRNA/mRNA ratio
age
D.
Male 10 days
Male 20 days
Male 30 days
Male 40 days
Male 50 days
Transcription (intron signal) Fold Change
n_neighbors
circRNA Fold Change (vs 0 days)
E.
Males (50 days old)
CLUSTER 1
CLUSTER 2
CLUSTER 3
CLUSTER 4
CLUSTER 5
Transcription (intron signal) FC
circRNA Fold Change (vs 0 days)

Figure S3.

Figure S3 circRNA expression follows three main patterns as fly ages. A. Heatmap displaying the Z-scores derived from pairwise statistical comparisons of key genomic features across circRNA clusters in male and female flies. Statistically significant differences are marked with asterisks (***, **, * denote adjusted p-values < 0.001, < 0.01, and < 0.05, respectively). **B.** Boxplot of the circRNA/mRNA ratios for each timepoint in males using all mRNA seq reads obtained by STAR alignment. **C.** Boxplot of circRNA/mRNA ratios for each timepoint in males using only junction reads obtained in *find_circ2*.. **D.** Scatter plots for each timepoint in males showing the fold changes in transcription (measured as total intron signal for the gene) *vs* circRNA levels compared to the first aging timepoint. **E.** Scatter plots of the 50-day timepoint in males for each circRNA cluster, showing fold changes in transcription *vs* circRNA levels compared to the first timepoint. **Related to Figure 4.**

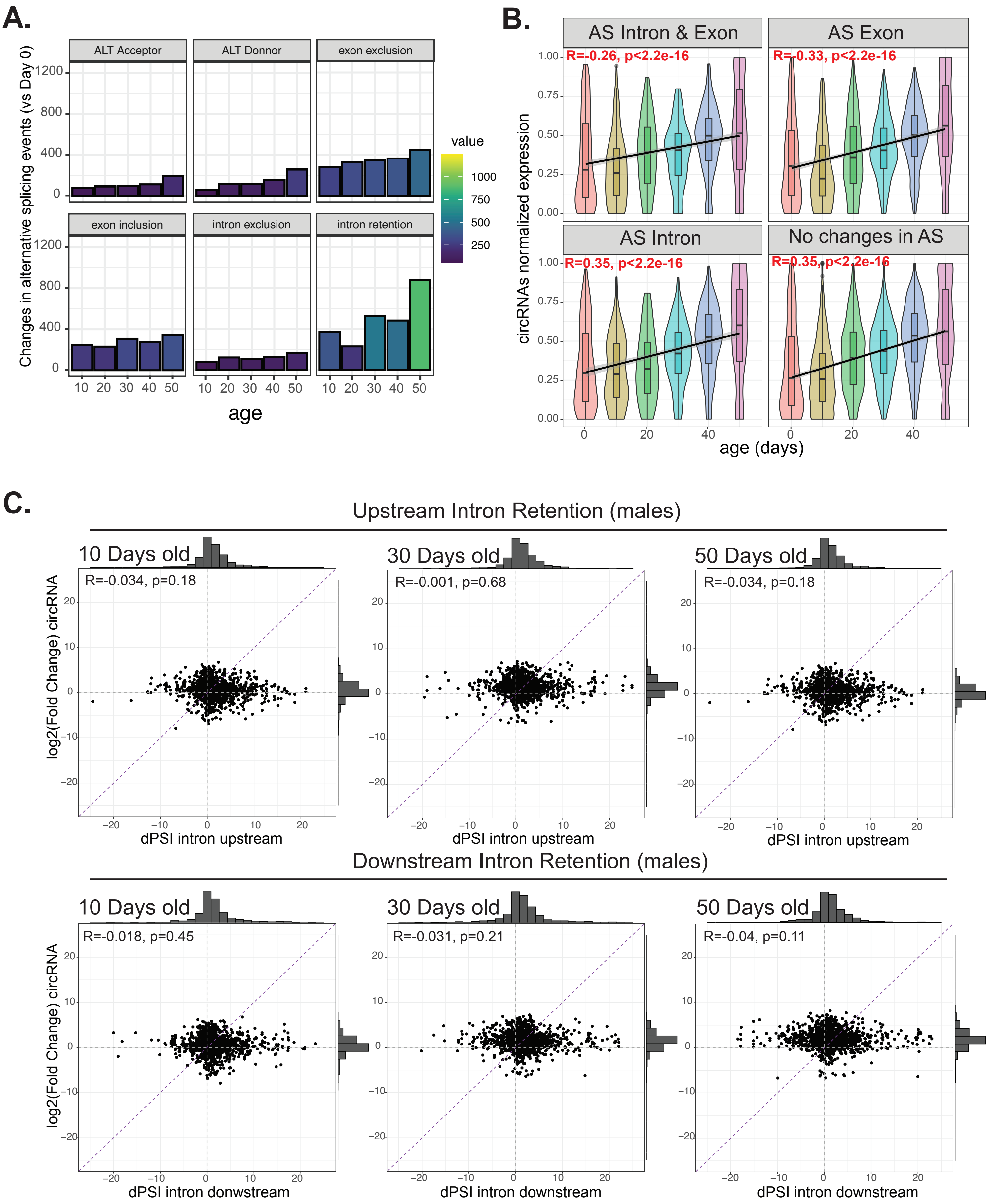

A.
ALT Acceptor
ALT Donnor
exon exclusion
exon inclusion
intron exclusion
intron retention
Changes in alternative splicing events (vs Day 0)
age
value
B.
AS Intron & Exon
AS Exon
AS Intron
No changes in AS
R=-0.26, p<2.2e-16
R=-0.33, p<2.2e-16
R=0.35, p<2.2e-16
R=0.35, p<2.2e-16
circRNAs normalized expression
age (days)
C.
Upstream Intron Retention (males)
10 Days old
30 Days old
50 Days old
R=-0.034, p=0.18
R=-0.001, p=0.68
R=-0.034, p=0.18
log2(Fold Change) circRNA
dPSI intron upstream
Downstream Intron Retention (males)
R=-0.018, p=0.45
R=-0.031, p=0.21
R=-0.04, p=0.11
dPSI intron donwstream
dPSI intron downstream

Figure S4.

Figure S4. Alternative splicing changes do not primarily drive circRNA accumulation with age. A. Number of alternative splicing events differentially regulated with age in male flies. The parameters of splicing analysis tested were: alternate splice acceptor, alternate splice donor, exon exclusion, exon inclusion, intron exclusion, and intron retention. **B.** Boxplot and Violin plot of normalized circRNA expression levels, divided into panels based on the presence (or absence) and type of changes in alternative splicing of their host gene. **C.** Correlation plot in males between circRNA fold change and ∂PSI for upstream (top panels) or downstream introns (bottom panels) at ages 10, 30, or 50 *vs*. age 0. **Related to Figure 5.**

A.

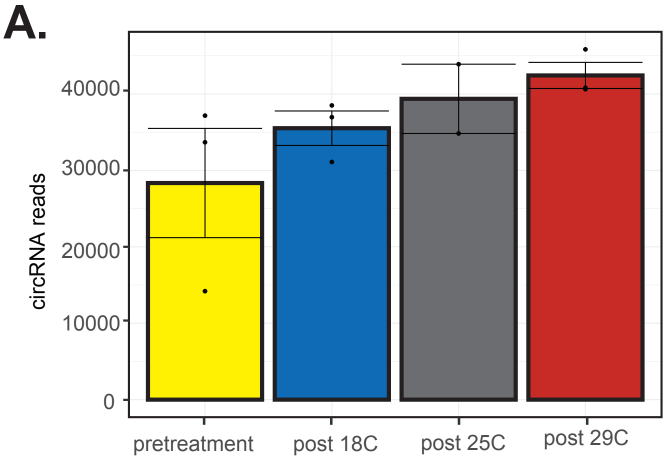

C.

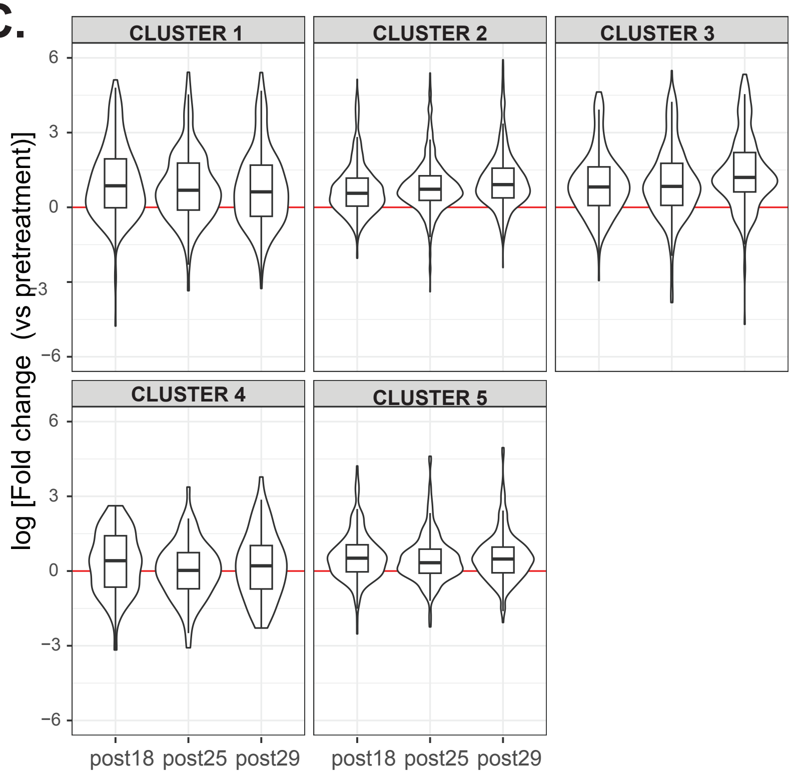

B.

	1928 circRNAs		13804 mRNAs	
	down	up	down	up
pre25 vs post18	4	151	730	674
pre25 vs post25	0	105	447	439
pre25 vs post29	6	316	1097	864
post18 vs post29	46	139	812	839

D.

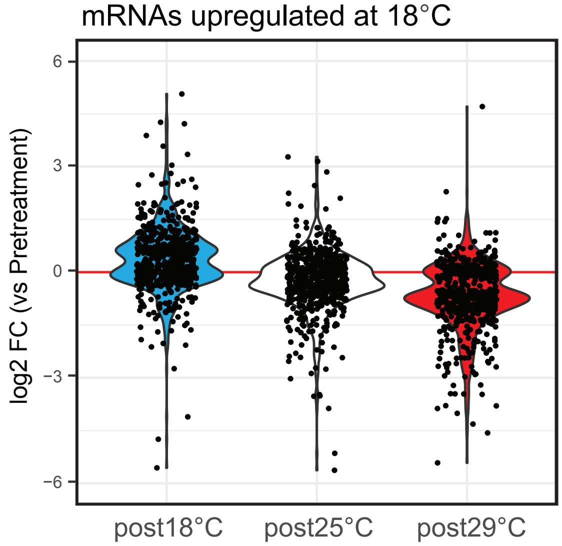

E.

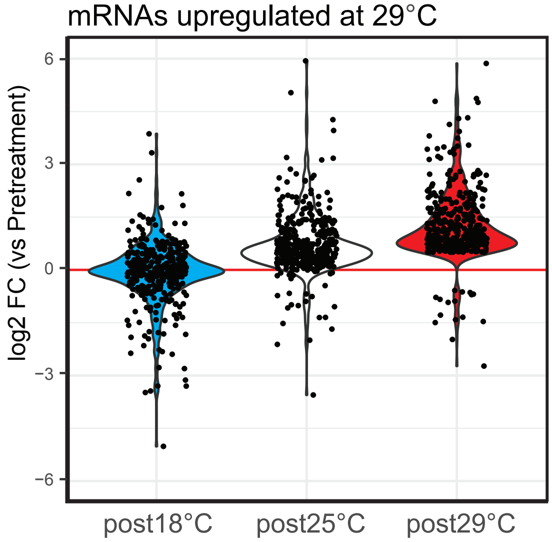

F.

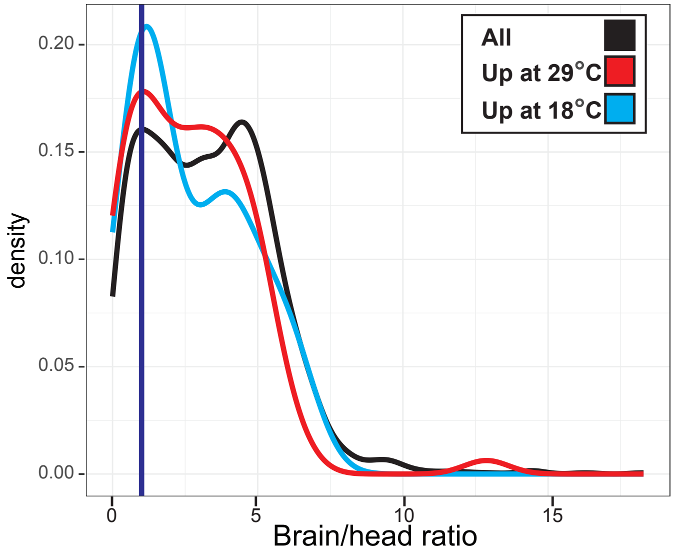

G

Upregulated at 29°C

Cell type	Max. Enrich	adj p
Photoreceptor-like	18.4	2 x 10-14
Visual System Neurons	18.4(35)	2 x 10-14
Octapaminergic Neurons	13.8	4 x 10-9
Mushroom body neurons	12.6 (4)	7x10-8
Antennal Projection Neurons	13.8	1x10-6
Dopaminergic PAM Neurons	11.5	4 x 10-9
Ocellus Retinula Cell	9.2	1.5x10-3
Poxn Neurons	8	1.5x10-3
Outer Photoreceptor cells	7	0.016

Upregulated at 18°C

Cell type	Max. Enrich	adj p
Mushroom body neurons	20(2)	2.6x10-6
Visual System Neurons	178)	6x10-4

Figure S5.

Figure S5. A subset of circRNAs increase their levels in response to temperature treatment. A. Total number of backsplicing reads on each condition is plotted as means ± SEM. **B.** The table indicates the number of circRNAs or mRNAs differentially expressed between the indicated conditions, with FDR<0.05, log2FoldChange > 0.5 or < - 0.5. **C.** The log fold changes of circRNAs in each of the five clusters are presented for each condition. **D.** Violin plot showing the log fold change of mRNAs upregulated at 18°C across all temperatures compared to the pretreatment sample. **E.** Violin plot illustrating the log fold change of mRNAs upregulated at 29°C across all temperatures compared to the pretreatment sample. **F.** Histogram illustrating the distribution of brain enrichment values for all differentially expressed circRNAs upon temperature treatment (in black) or those upregulated at 18 or 29 °C (in blue and red, respectively). The blue line marks the 1:1 brain-to-head ratio threshold. **G.** Summary table presenting the cell-type enrichment analysis for genes upregulated at 29 °C (top table) or 18 °C (bottom table) using the Cell Marker Enrichment tool. Cell types significantly enriched (corrected p-values <0.05) are listed. The maximum enrichment and adjusted p-value for each cell type are reported. Similar neuron/tissue types are summarized in one cluster, with average enrichment reported and the number of enriched cell types in brackets. **Related to Figure 6.**

A.

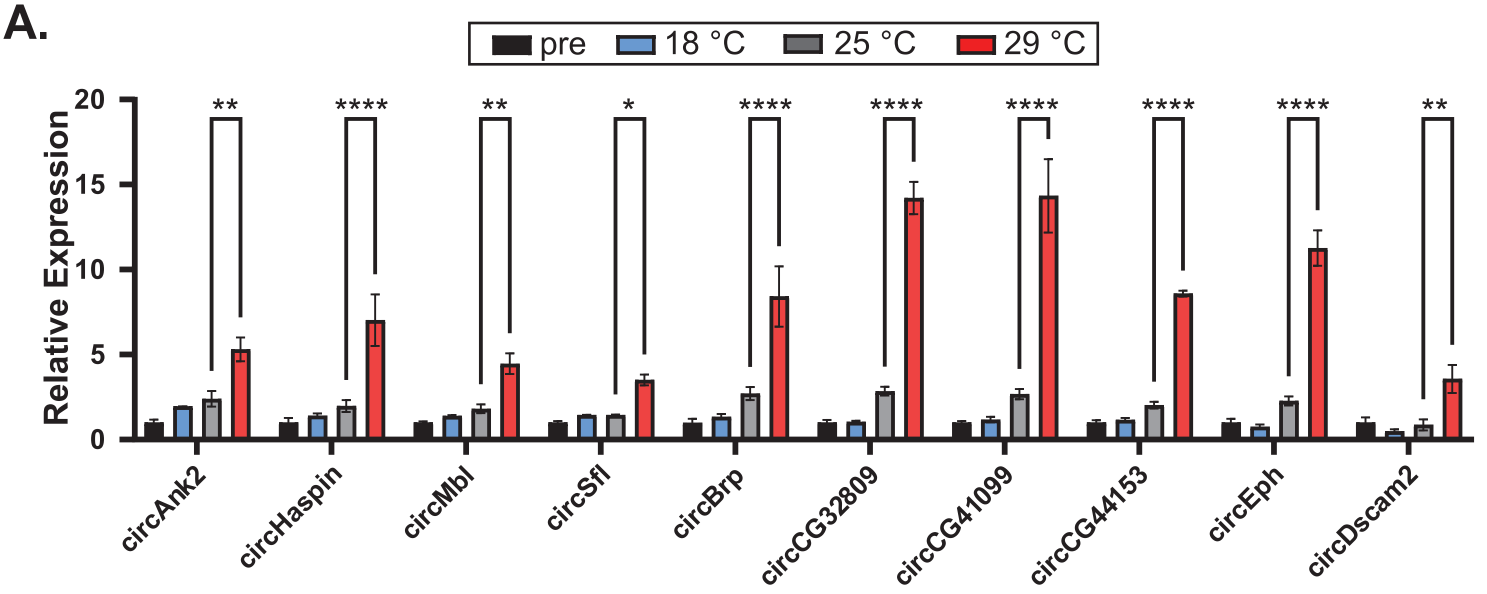

B.

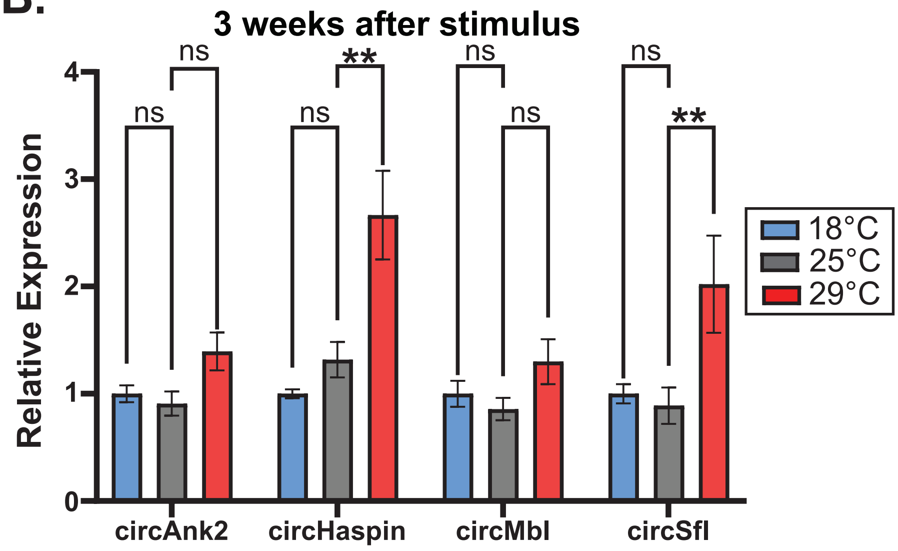

C.

circRNA	Approx. Half-life
circAnk2	>10 days
circHaspin	~37 days
circMbl	~14 days
circSfl	>60 days
circBrp	~12 days
circCG32809	>15 days
circCG41099	~15 days
circCG44153	~27 days
circEph	>60 days
circDscam2	~16 days

D.

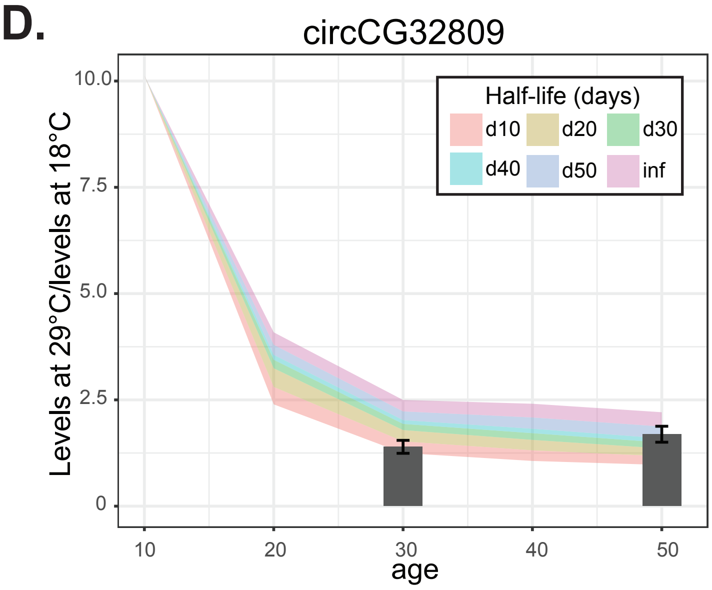

E.

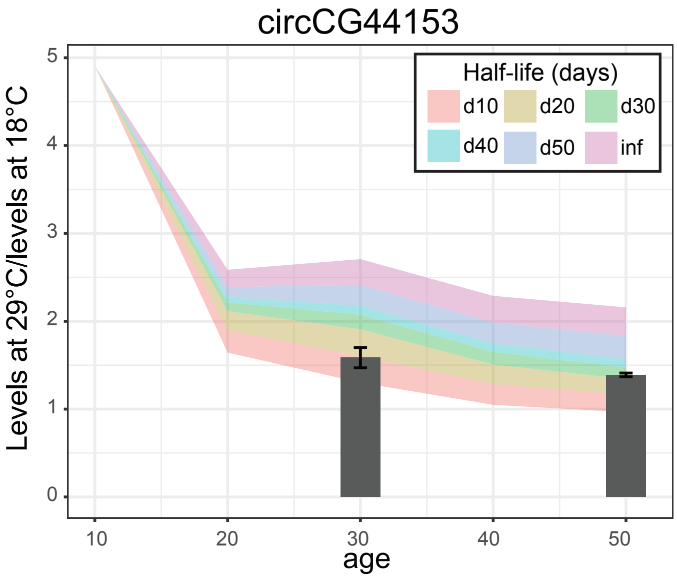

Figure S6.

Figure S6. circRNAs can be used as life experience markers. A. Relative expression of the indicated circRNAs in males after temperature treatment (N=3). **B.** Relative expression of the indicated circRNAs in males after three weeks of recovery from the temperature treatment (N=3-5). **C.** The table lists the estimated half-lives of the indicated circRNAs, obtained by averaging the estimated half-lives calculated using RT-qPCR data at 3 and 6 weeks. For circAnk2 and circCG32809 (in red) the two values were highly divergent. **D-E.** Fold change between 18°C and 29°C is modeled on aging data for circCG32809 (D) and circCG44153(E). Colored bands indicate projected fold change for each half-life, while bars represent experimental RT-qPCR fold change. **Related to Figure 6.**

Table S1. Statistic of alignment for aging RNAseq samples

Sample Name	Total Reads (millions)	Aligned Linear Reads	circRNA Reads	% circ/linear reads	Million aligned reads/timepoint
CS_F_0_1	96	79545496	35893	0.04514297	
CS_F_0_2	34.7	28862533	14337	0.04969808	
CS_F_0_3	102.4	84915875	36534	0.04304228	193.3
CS_F_10_1	71.1	58047909	41485	0.07151794	
CS_F_10_2	71.7	58506244	41893	0.07165563	
CS_F_10_3	62	51209569	36584	0.07149085	167.8
CS_F_20_1	116.4	95026968	73366	0.0772651	
CS_F_20_2	68	55412796	46613	0.08419038	
CS_F_20_3	63.2	51516423	45113	0.08764689	202
CS_F_30_1	70.6	57722809	53205	0.09225831	
CS_F_30_2	104.1	85299190	76691	0.08998915	
CS_F_30_3	58.4	47798648	47955	0.10042786	190.8
CS_F_40_1	62.6	51284349	62442	0.12190487	
CS_F_40_2	64.9	53236729	65094	0.12242242	
CS_F_40_3	89.6	73433691	83434	0.11374739	177.9
CS_F_50_1	67.1	55380161	69184	0.12508186	
CS_F_50_2	60.4	49757909	69184	0.13923481	
CS_F_50_3	64.6	53218726	70617	0.13286832	158.4
CS_M_0_1	92.5	76531270	35640	0.0465909	
CS_M_0_2	79.7	66040656	32790	0.0496759	
CS_M_0_3	78.4	64694875	30765	0.04757662	207.3
CS_M_10_1	51.4	42078885	26962	0.06411597	
CS_M_10_3	70.9	57649720	43033	0.0747014	99.7
CS_M_20_1	66.8	58379862	58130	0.09967125	
CS_M_20_2	78.4	64284466	60922	0.09485929	122.7
CS_M_30_1	70.2	57291987	65842	0.1150558	
CS_M_30_2	75.4	61234663	70810	0.11577099	
CS_M_30_3	65.9	53792244	65477	0.12187035	172.3
CS_M_40_1	54.6	46084124	65736	0.14284725	
CS_M_40_2	67.6	55062904	75277	0.13689807	
CS_M_40_3	68.9	56254568	77543	0.1380333	157.4
CS_M_50_1	65.8	53846306	78907	0.14675622	
CS_M_50_2	86.1	70396354	100948	0.1436054	124.2
				AVERAGE mR	164.4833333

Table S3. Main age determinants for circRNAs. PC1 from the PCA plotted in Figure 2B

	circRNA	Dim.1	
1	circ:chr3L:9771654:9781218:FBgn0036043	1.423654772	
2	circ:chr3R:19887071:19887951:FBgn0002940	1.153109816	
3	circ:chrX:11397824:11398822:FBgn0001624	1.132436068	
4	circ:chr3L:10584815:10585539:FBgn0052062	0.685712498	
5	circ:chr3R:17966472:17967828:FBgn0263995	0.677513941	
6	circ:chr3L:7662470:7663324:Ank2	0.630560409	
7	circ:chr2R:17275409:17276063:FBgn0265487	0.603698333	
8	circ:chr3L:22546930:22547938:intergenic	0.535356705	
9	circ:chr3R:20992240:20994423:FBgn0013995	0.534791295	
10	circ:chr3L:5703924:5706573:Sif	0.529914068	
11	circ:chr3L:7661980:7662890:Ank2	0.525294695	
12	circ:chr2R:13185783:13187908:ambiguous	0.477718378	3 genes overlapp: Kdm4B/CG17724/seq
13	circ:chr2L:10126000:10127877:FBgn0265002	0.456854591	
14	circ:chr4:938331:939117:FBgn0019985	0.4526706	
15	circ:chr3L:10568128:10568605:FBgn0052062	0.447596358	
16	circ:chr2L:18539522:18543715:FBgn0085370	0.443119016	
17	circ:chrX:7645779:7646979:FBgn0004198	0.438184415	
18	circ:chr2R:13180989:13183234:ambiguous	0.435562392	3 genes overlap: Kdm4B/CG17724/seq
19	circ:chr3R:14344224:14345184:FBgn0024321	0.431125682	
20	circ:chr3L:23126199:23128370:FBgn0053217	0.426704122	
21	circ:chr2R:22555217:22556794:FBgn0003175	0.415830576	
22	circ:chr2R:15452856:15456815:FBgn0263980	0.414234361	
23	circ:chr3R:9445043:9446497:FBgn0261552	0.412404002	
24	circ:chr2R:7602431:7606244:FBgn0033196	0.411781607	
25	circ:chr4:1164881:1166852:FBgn0039925	0.394273107	
